# Cross-omics mapping of the human HIV restrictome across clinical states, immune-cell compartments, and protein responses

**DOI:** 10.1371/journal.pone.0356591

**Published:** 2026-10-13

**Authors:** Reuben S. Maghembe

**Affiliations:** 1 Department of Microbiology and Parasitology, Faculty of Medicine, St. Francis University College of Health and Allied Sciences, Ifakara, Tanzania; 2 Department of Omics and Computational Biology, AfroBiomics Co. Ltd., Dar es Salaam, Tanzania; Albany Medical College, UNITED STATES OF AMERICA

## Abstract

Host restriction factors can block HIV at multiple stages, but they are often studied as individual genes or within a single molecular layer. I performed an evidence-structured computational reanalysis of three independent public datasets to determine how the human HIV restrictome varies across clinical states, immune-cell compartments, and protein responses. The analysis used bulk monocyte RNA sequencing (GSE157198), PBMC single-cell/CITE-seq data (GSE256089), and primary CD4 T-cell proteomics (PXD012263) within a prespecified restrictome-immunometabolism framework. In GSE157198, ART-naive monocytes had higher core-restrictome activity than healthy controls (difference = 1.154, p = 0.000746), and core scores were strongly correlated with interferon/innate-sensing scores (Spearman r = 0.800, p = 7.47 × 10^-11). Core-lipid/cholesterol correlation was weaker and was not independent of interferon after conditional analysis. In GSE256089, participant-paired core-restrictome scores were enriched in NK cells relative to B cells, monocytes, and T cells, and matched random-gene-set analyses supported enrichment beyond background expression structure. FCGR3A monocytes also had higher participant-paired core scores than CD14 monocytes. Exploratory sensing/death-gene analysis identified cell-type-specific expression patterns but did not establish pathway activation, pyroptosis, or apoptosis. In PXD012263, the historical q < 0.05 proteomic selection contained 19 curated genes and retained mixed response polarity; sensitivity thresholds q < 0.01 and q < 0.10 selected 10 and 25 genes, respectively. Together, the results show that HIV restrictome activity varies across clinical and immune-cell contexts and is embedded within broader interferon-linked and immunometabolic states. Because the datasets are independent and not subject matched, the findings are observational and hypothesis-generating rather than causal.

## Introduction

Antiretroviral therapy has transformed HIV infection into a manageable chronic condition, but it does not eliminate persistent viral reservoirs or the inflammatory and immune disturbances that accompany infection [1]. HIV also evolves rapidly through error-prone reverse transcription, recombination, and immune selection, generating extensive diversity within and among infected people [2–7]. These features make HIV persistence a problem of both viral adaptation and host-cell state.

Intrinsic restriction factors are host proteins that block HIV at entry, reverse transcription, nuclear import, integration, transcription, assembly, release, or cell-to-cell spread [8–11]. Established systems include APOBEC3 enzymes, SAMHD1, BST2/tetherin, TRIM proteins, MX2, IFITM proteins, SERINC3/SERINC5, MOV10, ZC3HAV1, and wider interferon-stimulated effector programs [9,10,12–18]. Many were defined through viral countermeasures: Vif antagonizes APOBEC3 proteins, Vpu targets BST2, Vpx counteracts SAMHD1 in HIV-2 and related lentiviruses, and Nef excludes SERINC3/SERINC5 from virions [14,15,17–20]. The restrictome therefore reflects an evolutionary contest between host antiviral pressure and viral counter-restriction.

Restriction factors do not operate as an interchangeable antiviral list. Their abundance and activity vary with leukocyte lineage, interferon exposure, activation, and metabolism [16,21–26]. APOBEC3 expression differs among tissues and hematopoietic subsets [27], TRIM5alpha illustrates lineage-specific restriction pressure [28], IFITM proteins exert context-dependent effects at cellular membranes [12], and interferon-stimulated gene screens distribute antiviral activity across many effectors [16]. Chronic HIV infection is also accompanied by systemic inflammation [29] and major immunometabolic remodeling [25]. Glucose and glutamine metabolism can support early infection in CD4 T cells [21], and highly metabolic CD4 T cells are preferentially susceptible to reservoir seeding [26]. Comorbidities such as diabetes and tuberculosis further alter inflammatory and metabolic environments relevant to HIV host responses [20,30–32].

Public HIV omics datasets now permit this biology to be examined across molecular and cellular scales, although the most informative datasets were generated for different primary questions [33–35]. GSE157198 investigated CREM/ICER isoform expression in spontaneous HIV control [35], GSE256089 identified monocyte programs associated with HIV-1 reservoir size after acute treatment [33], and PXD012263 mapped proteome dynamics during HIV infection of primary CD4 T cells [34]. Reanalyzing these resources through a common biological framework can therefore test whether signals separated by study design converge on a shared organization.

Here I asked whether the human HIV restrictome behaves as a uniform interferon-induced program or instead varies with clinical state, immune-cell identity, and molecular context. I analyzed bulk monocyte RNA sequencing, PBMC single-cell/CITE-seq, and primary CD4 T-cell proteomics as three complementary public evidence layers rather than as a subject-matched multi-omics cohort. This evidence-structured computational reanalysis tests three related questions: whether clinical state is associated with coordinated restriction activity, which immune-cell compartments carry these programs, and how selected restriction-related proteins behave during HIV exposure. The data suggest that immune cells deploy context-specific combinations of antiviral defenses rather than a single uniform intrinsic antiviral program.

## Results

### A common framework separates clinical state, cell compartment, and protein response

The analysis integrated three independent public omics datasets within one prespecified biological framework (Fig 1). GSE157198 contributed clinical-state-resolved monocyte transcriptomes, GSE256089 contributed participant-level immune-cell localization, and PXD012263 contributed protein responses in HIV-exposed primary CD4 T cells [33–35]. Because these datasets differ in cohort, cell type, and experimental design, they were treated as complementary evidence layers rather than as subject-matched multi-omics measurements. The framework retained the eight prespecified modules spanning core HIV restriction, interferon/innate sensing, nucleotide metabolism, lipid/cholesterol metabolism, mitochondrial/redox biology, glycolysis/inflammatory metabolism, autophagy/proteostasis, and immune-cell state. Reviewer-requested analyses were used as explicit robustness or exploratory extensions and did not retrospectively redefine the prespecified modules.

**Fig 1:**
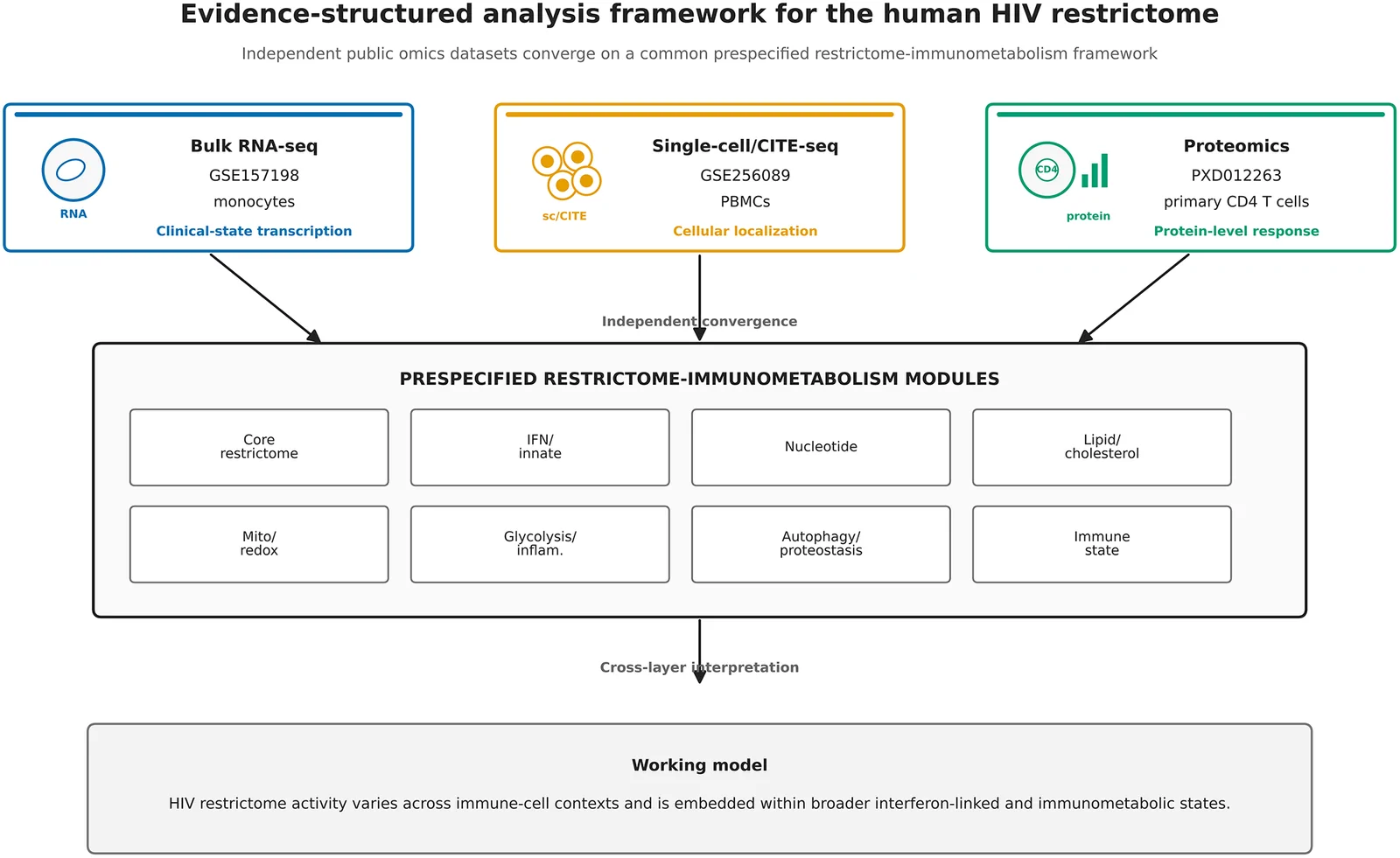
Evidence-structured analysis framework for the human HIV restrictome. Three complementary public omics datasets were analyzed as independent evidence layers: bulk monocyte RNA-seq from GSE157198 for clinical-state transcriptional differences, PBMC single-cell/CITE-seq from GSE256089 for cellular localization, and primary CD4 T-cell proteomics from PXD012263 for protein-level responses. Each dataset was interpreted through the same prespecified restrictome-immunometabolism framework comprising core restriction, IFN/innate, nucleotide, lipid/cholesterol, mitochondrial/redox, glycolysis/inflammatory metabolism, autophagy/proteostasis, and immune-state modules. The datasets are complementary evidence layers; subject-level multi-omic matching is not implied. The working model therefore treats restrictome activity as varying across immune-cell contexts and as embedded within broader interferon-linked and immunometabolic states rather than as a uniform or independently coupled program.

Table 1 summarizes the role of each dataset, and Table 2 defines the eight biological modules. The design separated three questions that are often conflated: whether clinical state changes coordinated restriction programs, which immune-cell compartments carry those programs, and whether transcript-level organization is preserved or remodeled at the protein level.

**Table 1:** Public datasets used in the study.

Dataset	Omics layer	Biological context	Role in this study	Main result
GSE157198	Bulk RNA sequencing	Enriched monocytes from healthy controls, ART-treated participants, untreated participants, and elite controllers	Disease-state transcriptomic layer	Untreated monocytes showed the strongest IFN-coupled core restrictome state; elite-controller monocytes were not uniformly elevated.
GSE256089	Single-cell/CITE-seq	PBMCs from 38 HTO-demultiplexed A5354 participants during ART	Cell-type localization and participant heterogeneity	Participant-level analysis showed specific NK core-restrictome enrichment beyond matched random gene sets and higher core-restrictome scores in FCGR3A than CD14 monocytes; broad reservoir-category module differences remained non-significant after correction.
PXD012263	Proteomics	Primary human CD4 T cells across an HIV infection time course	Protein-level remodeling layer	Selected restriction factors were depleted in HIV-positive cells while other antiviral and IFN-linked proteins were induced.

**Table 2:** Prespecified restrictome-immunometabolism module framework.

Module	Number of curated genes	Representative genes
Core HIV restriction factors	37	APOBEC3A; APOBEC3B; APOBEC3C; APOBEC3D; APOBEC3F; APOBEC3G; APOBEC3H; BST2; CH25H; DDX58; DDX60; HERC5
Interferon/innate sensing	25	CGAS; IFNAR1; IFNAR2; IFNB1; IFNGR1; IFNGR2; IKBKE; IRF1; IRF3; IRF7; IRF9; JAK1
Nucleotide metabolism	14	CAD; DCK; DHFR; GMPS; IMPDH1; IMPDH2; MTHFD1; MTHFD2; RRM1; RRM2; RRM2B; SAMHD1
Lipid/cholesterol membrane metabolism	17	ABCA1; ABCG1; ACACA; CH25H; CPT1A; CPT2; FASN; HMGCR; HMGCS1; LDLR; NPC1; NPC2
Mitochondrial/redox biology	23	ATP5F1A; CAT; COX4I1; GCLC; GCLM; GPX1; GPX4; GSR; HMOX1; NDUFA1; NDUFB8; NFE2L2
Glycolysis/PPP/inflammatory metabolism	26	ACOD1; ALDOA; ARG1; ARG2; ENO1; G6PD; GAPDH; GPI; HIF1A; HK1; HK2; IDO1
Autophagy/proteostasis/ER stress	22	ATF4; ATF6; ATG12; ATG16L1; ATG3; ATG5; ATG7; BECN1; DDIT3; EIF2AK3; ERN1; HSP90AA1
Immune-cell state/activation/exhaustion	36	CCL2; CCL3; CCL4; CCR7; CD3D; CD3E; CD4; CD79A; CD8A; CD8B; CLEC9A; CTLA4

### ART-naive monocytes show a robust interferon-linked restrictome elevation

GSE157198 contained enriched monocyte transcriptomes from healthy controls, ART-treated individuals, untreated individuals, and elite controllers [35]. All 37 genes in the core HIV restriction module were detected, and most immunometabolic modules had high coverage, supporting module-level comparison across clinical groups.

The ART-naive group showed the strongest elevation in the core-restrictome score relative to healthy controls (difference = 1.153725, p = 0.000745751; Fig 2D). Across all four groups, the core-restrictome score showed a substantial group effect (eta2 = 0.420447; Kruskal-Wallis p = 0.00279821). The core-restrictome score was strongly correlated with the interferon/innate-sensing score (Spearman r = 0.799718, p = 7.46724 × 10^-11; Fig 2E). The core score also correlated with the lipid/cholesterol score in the unadjusted analysis (Spearman r = 0.451163, p = 0.00211408; Fig 2F). This association remained detectable after removing CH25H, which is shared by the core and lipid gene sets (r = 0.424242, p = 0.00410444), showing that simple gene-set overlap was not sufficient to explain the unadjusted correlation. However, the lipid association was not independent of interferon. After conditioning on the interferon/innate-sensing score, the partial correlation fell to r = 0.059104 (p = 0.703132); after conditioning on both interferon and clinical group, it was r = −0.017597 (p = 0.909734). The lipid/cholesterol signal is therefore interpreted as part of a shared IFN-associated immunometabolic state rather than as an independent core-lipid coupling axis. Across three prespecified null schemes containing 1,000 random 37-gene sets each, the ART-naive-versus-healthy effect had empirical p = 0.000999001 under every scheme (BH-adjusted empirical p = 0.002997). The core-IFN correlation was also robust, with empirical p values ranging from 0.000999001 to 0.001998002 (BH-adjusted values 0.002997–0.004995). By contrast, empirical p values for the unadjusted core-lipid relationship ranged from 0.0239760 to 0.0989011, and the IFN-adjusted core-lipid association remained null (empirical p = 0.700300–0.715285). Thus, the disease-state and core-IFN signals were robust to random-set testing, whereas lipid specificity was weaker. In addition, the ART-naive-versus-healthy disease-effect direction remained positive in all 37 leave-one-core-gene-out analyses, indicating that the result was not dependent on a single dominant core-restrictome gene.

**Fig 2:**
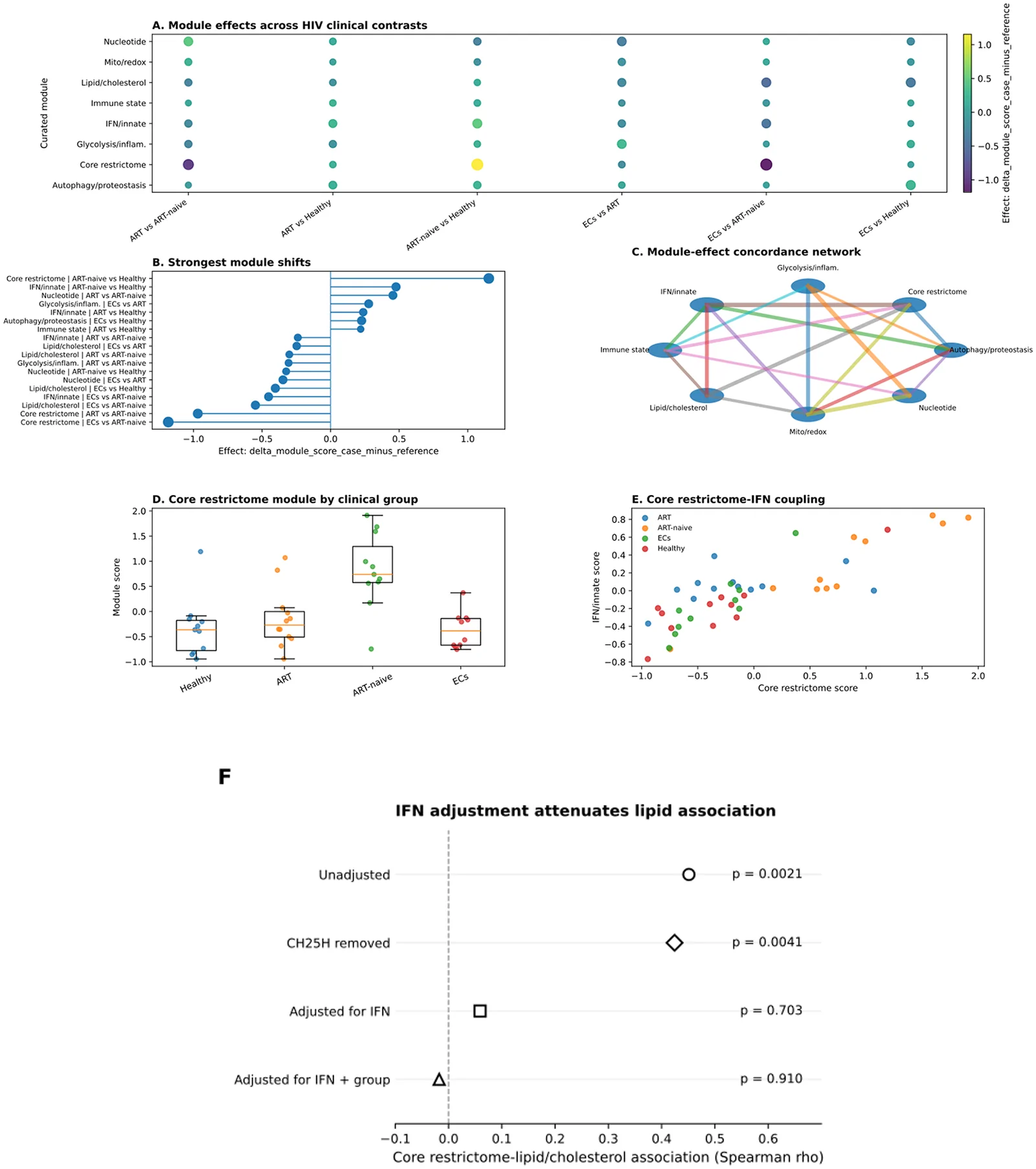
Bulk-monocyte restrictome and immunometabolic architecture in GSE157198. (A) Module effects across clinical contrasts. (B) Strongest module shifts. (C) Concordance of module effects. (D) Core-restrictome scores across clinical groups. The ART-naive-versus-healthy difference was 1.154 (p = 0.000746), and the four-group comparison gave eta2 = 0.420 and Kruskal-Wallis p = 0.00280. Across three prespecified 1,000-set random-gene null schemes, the ART-naive-versus-healthy effect had empirical p = 0.000999 in each scheme. (E) Core-restrictome and interferon/innate-sensing scores were strongly associated (Spearman r = 0.800, p = 7.47 × 10^-11). (F) Core-restrictome and lipid/cholesterol scores were associated in the unadjusted analysis (Spearman r = 0.451, p = 0.00211) and after removing the overlapping CH25H gene from the lipid score (r = 0.424, p = 0.00410), but the association was not independent of interferon: partial Spearman r = 0.059, p = 0.703 after conditioning on IFN and r = −0.018, p = 0.910 after conditioning on IFN plus clinical group. Lipid/cholesterol activity is therefore interpreted as part of a shared IFN-associated architecture rather than an independent core-lipid relationship. The ART-naive disease-effect direction remained positive in all 37 leave-one-core-gene-out analyses.

### Multiple antiviral families drive the monocyte state

Gene-level decomposition showed that the untreated-versus-healthy contrast was distributed across multiple antiviral families (Fig 3). The largest contributors included IFITM1, ISG15, RSAD2, MX1, IFITM3, HERC5, OAS3, LY6E, APOBEC3A, OAS2, MX2, DDX60, IFIH1, OAS1, IFITM2, TRIM22, PML, MOV10, and BST2.

**Fig 3:**
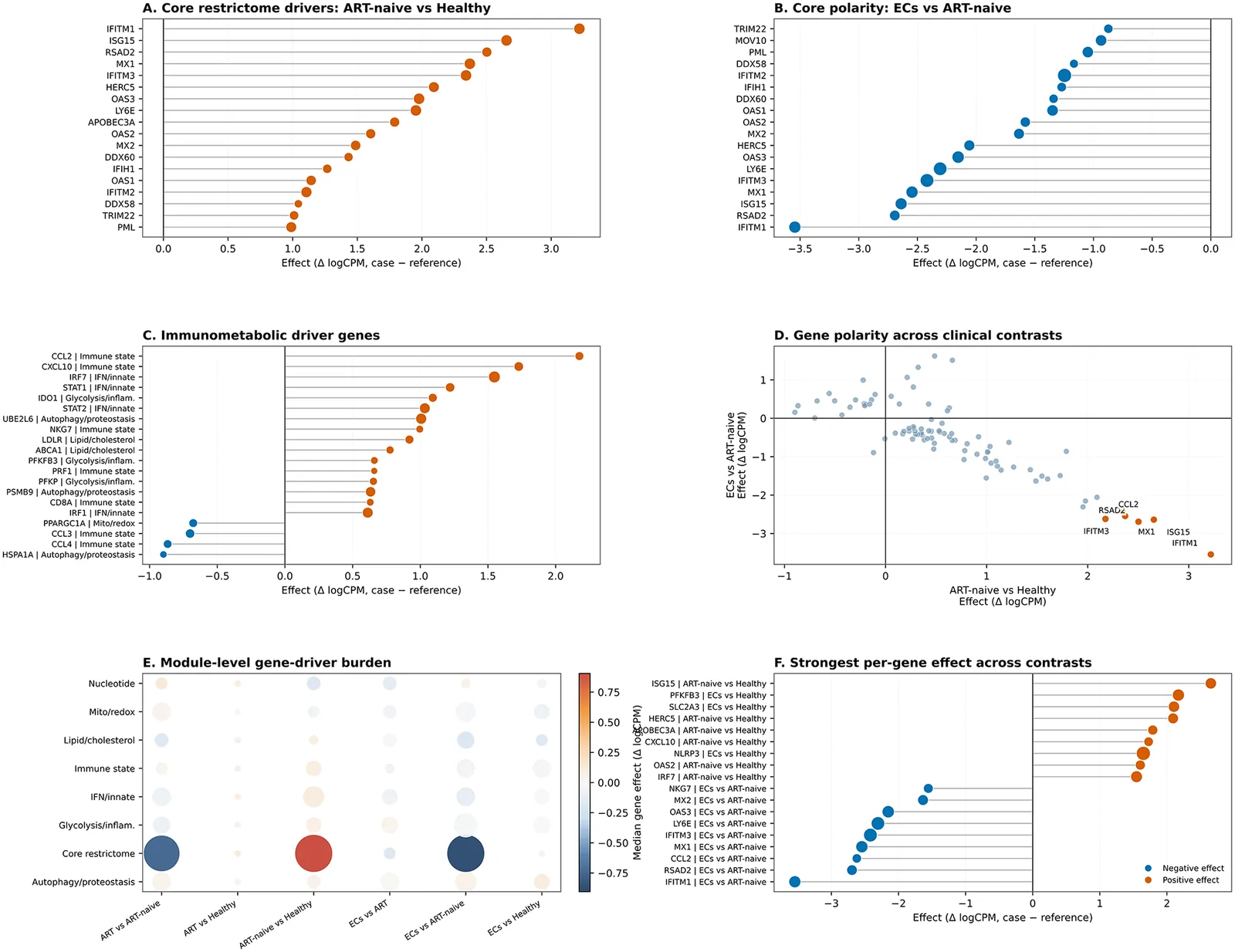
Gene-level drivers of monocyte restrictome and immunometabolic polarity in GSE157198. (A) Core restrictome genes with the largest absolute effects in ART-naive participants versus healthy controls. (B) Core restrictome polarity in elite controllers (ECs) versus ART-naive participants. (C) Non-core immunometabolic genes with the largest absolute effects in ART-naive participants versus healthy controls. (D) Two-contrast gene-polarity map for ART-naive versus healthy and EC versus ART-naive comparisons. All 90 historically displayed points are retained, while labels are restricted to 6 of the strongest unique genes to preserve readability. (E) Module-level gene-driver burden across clinical contrasts. Bubble size follows the existing analysis rule based on the number of genes with BH-adjusted p<=0.10, and color represents median gene-level effect. (F) Concise cross-contrast summary showing, for each gene, its single largest absolute effect among the existing clinical contrasts; the 18 strongest genes are displayed. Panels use the previously derived gene-level contrast table and do not refit models or recalculate multiple-testing correction. Gene-level lipid/cholesterol effects are shown as part of the broader immunometabolic architecture and are not interpreted as evidence of interferon-independent lipid coupling.

No single factor dominated the monocyte state. Instead, IFITM, OAS, MX, APOBEC3, ubiquitin-like modification, viral sensing, and virion-release pathways contributed in parallel. The elite-controller contrast remained weaker along the dominant interferon-restrictome axis, reinforcing the distinction between coordinated antiviral activation and durable control.

The leave-one-gene-out analysis supports this distributed interpretation: removing each core-restrictome gene in turn preserved the direction of the ART-naive-versus-healthy effect in 37 of 37 analyses. Thus, the clinical-state signal is not attributable to one dominant gene such as a single highly responsive interferon-stimulated factor.

### Participant-level single-cell analysis identifies specific NK enrichment and monocyte-subtype structure

GSE256089 provided HTO-demultiplexed PBMC profiles from 38 participants receiving ART in the A5354 cohort [33]. I used these data to localize the prespecified modules across broad immune-cell compartments and then linked participant-level scores to published reservoir categories.

Broad cell-type localization remained descriptive, but all inferential comparisons were performed using participants rather than individual cells as the independent units (Fig 4). Across participants, the mean core-restrictome score was 0.395507 in NK cells, 0.323249 in monocytes, 0.309693 in T cells, and 0.238208 in B cells. The participant-paired difference between NK cells and each participant’s mean across B cells, monocytes, and T cells was 0.105124 (dz = 3.8490; paired p = 5.551754 × 10^-24), and NK cells had the highest core score in 35 of 38 participants. Because lineage-specific baseline expression could generate an apparent NK-high pattern, I compared the observed enrichment with 1,000 random gene sets per prespecified matching scheme. These sets matched the core module for gene-set size and expression/detection structure. The observed NK-versus-reference enrichment remained unusual under the matched null distributions, with frozen empirical upper-tail p values of 0.001998, 0.007992, and 0.000999 across the relevant schemes. In contrast, simple counting of participants in which NK cells ranked highest was less specific under matched nulls. The result is therefore interpreted as quantitative enrichment of the curated restrictome beyond lineage background, not merely as an NK-high ranking. Higher-resolution monocyte analysis identified additional structure. Across 38 participants, 37,081 CD14 cells and 7,581 FCGR3A cells met the analysis criteria. Participant-paired core-restrictome scores were higher in FCGR3A than CD14 monocytes by 0.157650 on average (dz = 2.95165; paired p = 4.773127 × 10^-20), with FCGR3A exceeding CD14 in 37 of 38 participants. The participant-level relationship between monocyte composition and core-restrictome activity was also positive (Pearson r = 0.634, p = 1.92 × 10^-5; Spearman r = 0.614, p = 4.17 × 10^-5). The available single-cell annotations did not provide validated participant-linked CD56bright and CD56dim NK-cell labels. I therefore did not infer NK subtypes from unvalidated marker combinations.

**Fig 4:**
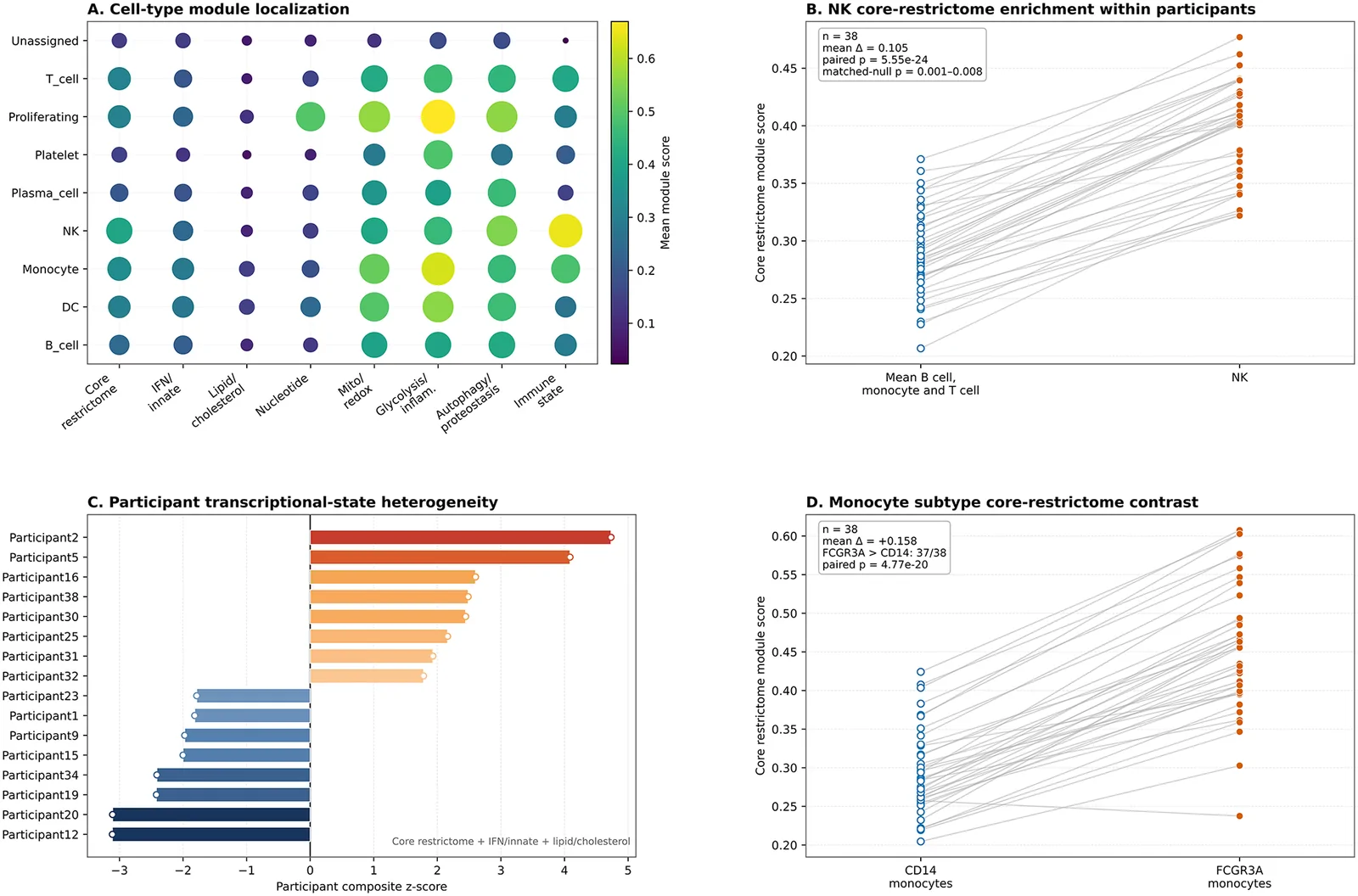
Single-cell localization and participant-level restrictome structure in GSE256089. (A) Mean scores for the eight prespecified modules across broad PBMC cell-type labels provide a descriptive localization overview. (B) Participant-paired comparison of the core-restrictome score in NK cells with each participant’s mean score across B cells, monocytes, and T cells. NK enrichment was observed across 38 participants (mean paired difference 0.105); matched random-gene-set analyses supported specificity beyond gene-set size and expression/detection structure. (C) Ranked participant-level transcriptional heterogeneity, showing the eight lowest and eight highest values of the prespecified core-restrictome + IFN/innate + lipid/cholesterol composite z-score. Color encodes signed direction and magnitude around zero and does not represent an additional statistical test. (D) Participant-paired comparison of core-restrictome scores between CD14 and FCGR3A monocyte subtypes. FCGR3A scores exceeded CD14 scores in 37 of 38 participants, with a mean paired difference of 0.158. All inferential comparisons use participants, not cells, as the independent unit.

### Monocyte composition does not explain the independent bulk clinical-state effect

Because FCGR3A and CD14 monocytes differed in restrictome activity, I tested whether variation in monocyte subtype balance could explain the GSE157198 clinical-state result. Three transcriptional proxies of CD14-FCGR3A balance were evaluated in the independent bulk-monocyte cohort. After adjustment, the ART-naive-versus-healthy core-restrictome effect remained approximately 1.14–1.17, with p values approximately 0.0006–0.0011. Monocyte subtype composition is therefore biologically associated with restrictome activity, but transcriptional proxies of CD14-FCGR3A balance did not account for the clinical-state-associated restrictome elevation observed in the independent GSE157198 bulk-monocyte cohort.

### Exploratory sensing and cell-death genes show context-specific expression without establishing pathway activation

cGAS and STING were already represented in the prespecified interferon/innate-sensing framework through MB21D1/CGAS and TMEM173. In response to the reviewer, I additionally examined IFI16, TREX1, CASP1, and CASP3 as exploratory targets. These additions were analyzed separately from the prespecified module tests. In the GSE157198 bulk-monocyte analysis, TREX1 showed clear FDR-supported variation, whereas the bulk cGAS/MB21D1 and STING/TMEM173 contrasts were not FDR-supported. In the participant-level GSE256089 analysis, IFI16, STING/TMEM173, and CASP3 showed higher expression in NK cells, whereas TREX1 and CASP1 were higher in monocytes. cGAS/CGAS did not differ materially between NK cells and monocytes. These expression patterns identify cell-context differences but do not establish cGAS-STING pathway activation, inflammasome activation, pyroptosis, executioner-caspase activity, or apoptosis. Functional activation of these pathways remains a mechanistic hypothesis requiring direct perturbational or biochemical testing.

### Primary CD4 T-cell proteomics shows mixed polarity that is stable to FDR-threshold sensitivity

PXD012263 provided a time-resolved proteomic map of HIV infection in primary human CD4 T cells [34]. I used the published source tables to ask whether proteins represented in the curated framework showed uniform induction or selective, bidirectional remodeling in HIV-positive cells.

The historical q < 0.05 WT-versus-mock selection remains the primary differential proteomic boundary. Among 137 curated genes with evaluable published q values, 19 met q < 0.05, reproducing the historical curated membership exactly (Fig 5C). The selected proteins showed mixed rather than uniformly induced polarity. This supports selective remodeling of restriction-related and interferon-linked proteins rather than a single-direction protein response. Sensitivity analysis using the same published q values selected 10 curated genes at q < 0.01, 19 at q < 0.05, and 25 at q < 0.10 (Fig 5D). Mixed polarity persisted, although the balance of increased, depleted, and stable/subtle proteins depended on threshold. The source-table time-course abundance summaries in Fig 5B and Fig 5E are descriptive overlays. They are not interpreted as independent time-course differential-significance tests. No quantitative protein-abundance imputation was identified in this reanalysis. Missing source-table abundance values were retained as missing. Replicate summaries used available nonmissing values, and a pseudocount of 1 was included in derived log2-ratio calculations. The pseudocount did not replace missing protein-abundance measurements and is distinct from missing-value imputation.

**Fig 5:**
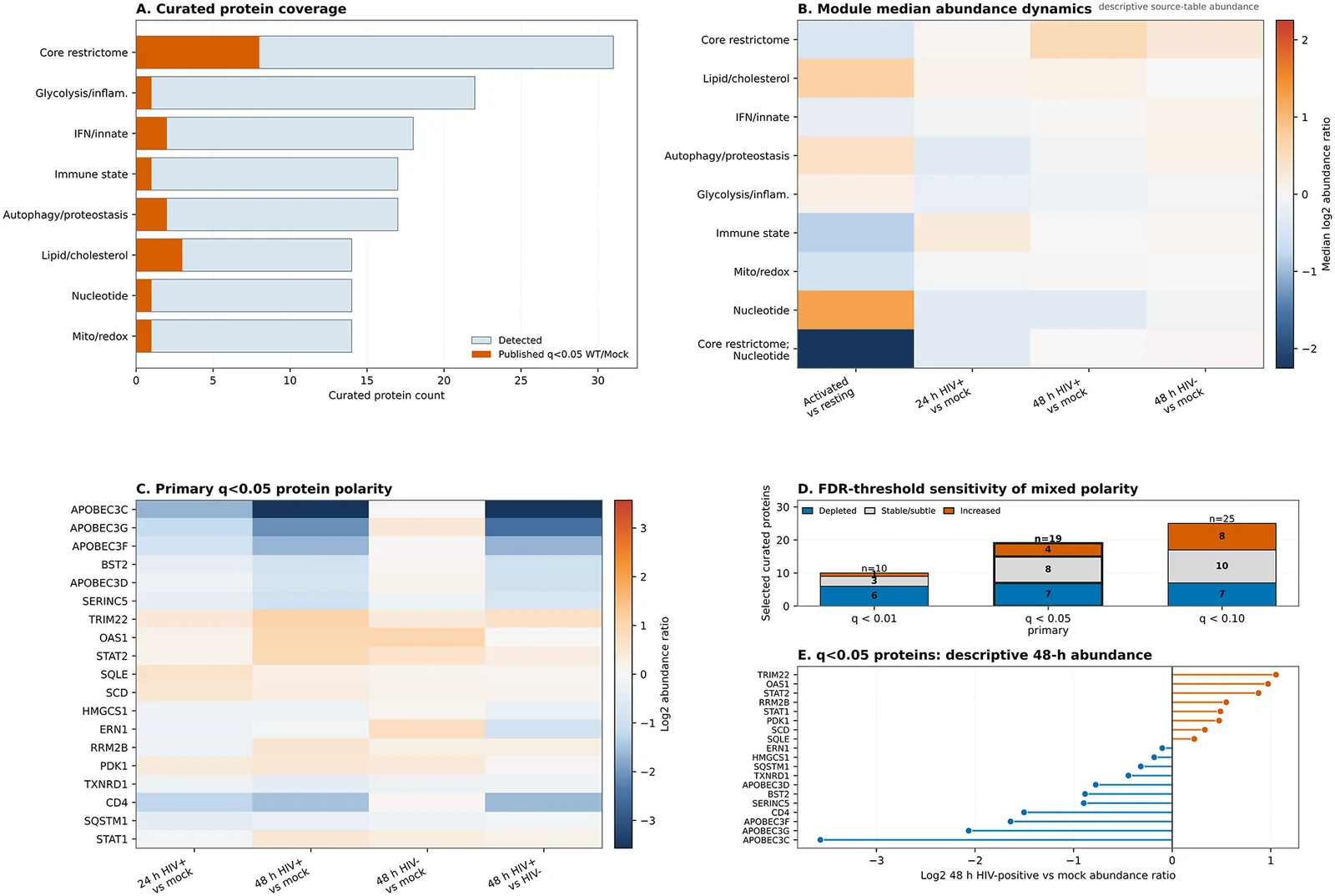
Proteomic overlay, primary q < 0.05 polarity, and FDR-threshold sensitivity in primary CD4 T cells. (A) Curated protein detection and the subset represented in the published WT-versus-mock q < 0.05 table. (B) Median source-table abundance dynamics across module-level comparisons; these time-course summaries are descriptive and are not interpreted as time-course differential-significance tests. (C) Abundance polarity of the 19 curated proteins in the historical published q < 0.05 WT-versus-mock selection, which remains the primary proteomic selection. (D) Sensitivity across published q < 0.01, q < 0.05, and q < 0.10 thresholds. Among 137 q-evaluable curated genes, 10, 19, and 25 were selected, respectively. The q < 0.05 set reproduced the historical 19-gene membership exactly. Mixed polarity persisted, although the balance of increased, depleted, and stable/subtle proteins was threshold-dependent. (E) Descriptive 48-h HIV-positive-versus-mock abundance direction for the same q < 0.05-selected proteins. The q threshold derives from the published single-timepoint source table; 48-h abundance is shown descriptively rather than as an independent significance test.

Across the three layers, the evidence converged on a model in which the HIV restrictome is organized by clinical state, immune-cell compartment, and selective protein remodeling (Fig 6; Tables 3 and 4). The integrated architecture distinguishes transcriptional activation from cell-type localization and from the fate of individual proteins in infected cells.

**Table 3:** Cross-layer evidence supporting the integrated model.

Evidence layer	Dataset	Main figure	Key support	Interpretation scope
Bulk monocyte RNA sequencing	GSE157198	Figs 2 and 3	Module- and gene-level contrasts identify an untreated high-IFN/high-restrictome monocyte state.	Associative monocyte host-state signal; not a direct infected-cell measurement.
Single-cell PBMC profiling	GSE256089	Fig 4	Restriction and immunometabolic modules localize to different PBMC cell types.	Reservoir-category differences were not robust after correction.
Primary CD4 T-cell proteomics	PXD012263	Fig 5	Curated proteins show both depletion and induction in HIV-positive cells.	Published source-table overlay; not raw mass-spectrometry reprocessing.
Integrated synthesis	All layers	Fig 6	Disease state, cell compartment, and protein polarity converge on one organizational model.	Cross-study convergence; not a causal or subject-matched trajectory.

**Table 4:** Genes and proteins supporting the integrated model.

Category	Representative genes/proteins	Primary source	Main figure/table
Bulk monocyte induced core/ISG drivers	IFITM1, ISG15, RSAD2, MX1, IFITM3, HERC5, OAS3, LY6E, APOBEC3A, OAS2, MX2, DDX60, IFIH1, OAS1	GSE157198	Fig 3; Table S4
Proteomic q < 0.05 HIV-positive-cell depleted restriction factors	APOBEC3C, APOBEC3G, APOBEC3F, APOBEC3D, BST2, SERINC5	PXD012263	Fig 5; Table S8
Proteomic q < 0.05 HIV-positive-cell induced proteins	TRIM22, OAS1, STAT2	PXD012263	Fig 5; Table S8
Proteomic lipid/cholesterol support	SQLE, SCD, HMGCS1	PXD012263	Fig 5; Table S8
High-dynamic non-q < 0.05 proteomic ISG/restrictome proteins	RSAD2, MX2, DDX60, ISG15, MX1, HERC5, OAS3, SP100, DDX58, IFIH1	PXD012263	Fig 5; Table S8

**Fig 6:**
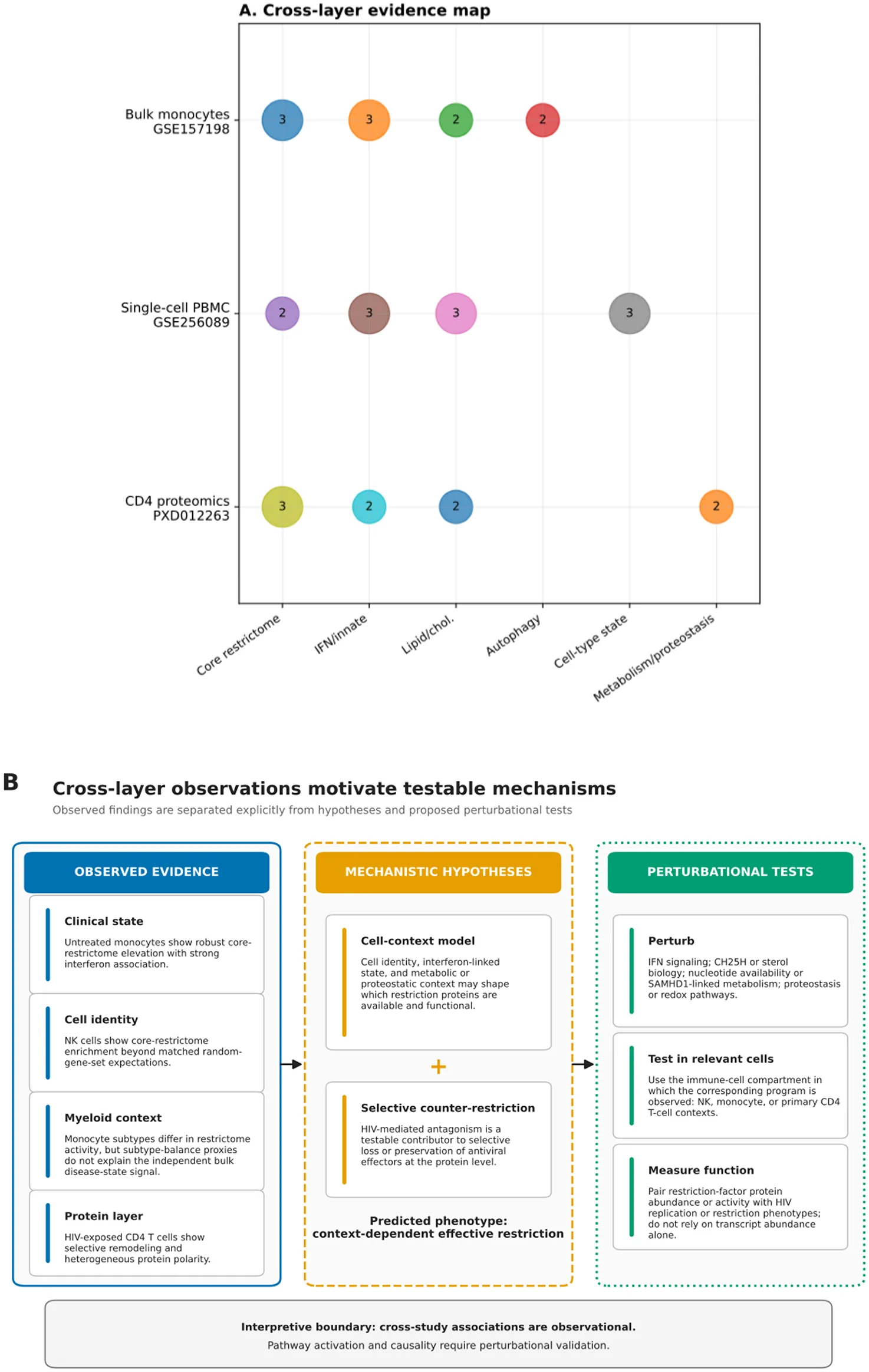
Evidence-structured working model and testable hypotheses for the human HIV restrictome. (A) Observed evidence from independent public datasets is summarized across three complementary layers: clinical-state-associated monocyte transcription in GSE157198, participant-level immune-cell localization in GSE256089, and protein responses in primary CD4 T cells in PXD012263. The datasets are not subject matched, and cross-layer convergence does not represent a within-person trajectory. (B) The observed patterns motivate noncausal, testable hypotheses in which interferon state, immune-cell lineage, monocyte subtype composition, lipid/cholesterol state, nucleotide availability, proteostasis/autophagy, and redox context may modulate restrictome expression or function. Hypothesis arrows represent proposed relationships for experimental testing rather than established causal paths. Candidate perturbational tests include manipulating cholesterol synthesis or trafficking, nucleotide availability, redox state, autophagy, or proteostasis while measuring restriction-factor abundance and antiviral function in defined immune-cell contexts. The framework therefore separates observed evidence, mechanistic hypothesis, and perturbational test and suggests that host-directed strategies should be evaluated in the cellular contexts in which the relevant restriction programs operate.

## Discussion

This evidence-structured computational reanalysis shows that the human HIV restrictome varies across clinical states, immune-cell compartments, and protein-response contexts. The strongest bulk-monocyte signal occurred in ART-naive HIV and was tightly associated with interferon/innate-sensing activity. At single-cell resolution, the curated core restrictome was quantitatively enriched in NK cells beyond matched background gene sets, while monocyte subtype was also associated with restrictome activity. In primary CD4 T-cell proteomics, selected restriction-related and interferon-linked proteins showed mixed response polarity rather than uniform induction. These observations support a context-dependent view of intrinsic antiviral defense but do not establish a causal or subject-matched molecular trajectory across the three independent datasets.

The monocyte findings refine the interpretation of spontaneous HIV control. Luo and colleagues identified CREM/ICER isoform differences in the same cohort [35], whereas the present framework resolved a broader interferon-coupled restrictome state. That state was strongest in untreated infection, not in elite control. Durable control therefore appears distinct from maximal peripheral monocyte interferon activation, consistent with evidence that HIV controllers rely on coordinated immune mechanisms rather than generalized activation [28,36,37] and with the contribution of chronic inflammation to HIV pathogenesis [29].

The gene-level pattern also explains why a module perspective is more informative than a single-factor ranking. IFITM, OAS, MX, APOBEC3A, RSAD2, ISG15, HERC5, DDX60, IFIH1, TRIM22, PML, MOV10, and BST2 jointly shaped the untreated monocyte state, consistent with broad host-factor and interferon-effector screens [16,19,33–35,38–40]. These factors act at different stages of the viral life cycle, including entry and fusion, nucleic-acid sensing, reverse transcription, post-entry trafficking, and virion release [13–18,22], so their coordinated elevation represents a composite antiviral state rather than one mechanism.

The single-cell layer separated the location of antiviral programs from their association with reservoir size. The source study identified monocyte expression profiles associated with HIV-1 reservoirs [33], whereas the broad modules used here localized core restriction activity most strongly to NK cells and interferon-lipid activity to monocytes and DCs. This division of labor offers a biological explanation for disagreement among bulk studies: the same pathway score can reflect altered cell composition, cell-intrinsic activation, or shared cytokine exposure.

The NK-cell result also requires a distinction between localization and induction. NK cells are developmentally equipped for rapid antiviral responses, so a high broad antiviral score could arise from constitutive lineage biology rather than HIV-specific activation. The matched-null analysis addresses part of this concern: the curated core-restrictome enrichment exceeded that expected from gene sets matched for size and expression/detection structure. I therefore interpret the signal as constitutive/developmental antiviral readiness together with specific restrictome enrichment, not as evidence that HIV uniformly induced the program in NK cells. The monocyte subtype analysis reveals a different source of cellular structure. FCGR3A monocytes had higher participant-paired core-restrictome scores than CD14 monocytes, and subtype composition correlated with participant-level restrictome activity. However, adjustment for transcriptional proxies of CD14-FCGR3A balance did not remove the independent ART-naive clinical-state effect in GSE157198. Cell composition can therefore modulate a restrictome measurement without being sufficient to explain the bulk disease-associated elevation. The myeloid localization findings are consistent with the broader biological importance of dendritic-cell and monocyte antiviral programs [41]. The lack of corrected reservoir-category differences is important because it defines the boundary of the module framework. It does not contradict the source study’s reservoir-associated signatures [33]; rather, it shows that broad, prespecified biological modules are better suited to mapping cellular architecture than to reproducing a dataset-specific biomarker model. This distinction prevents cell-type localization from being misinterpreted as reservoir prediction.

The exploratory sensing/death analysis adds another boundary to the interpretation. cGAS and STING were already represented in the prespecified innate module, while IFI16, TREX1, CASP1, and CASP3 were examined as reviewer-requested exploratory targets. Their expression differed across clinical or cell-type contexts, with TREX1 showing the clearest bulk statistical support and several targets showing NK- or monocyte-enriched single-cell expression. These observations identify candidate cellular contexts for follow-up but cannot establish DNA-sensing pathway activation or a specific mode of CD4 T-cell death. Direct assays of second-messenger production, inflammasome assembly, caspase cleavage, membrane integrity, or related functional endpoints would be required to distinguish pathway activation, pyroptosis, apoptosis, or compensatory expression [42,43].

The proteomic results expose a further asymmetry between antiviral signaling and restriction-factor abundance. The source atlas mapped extensive host remodeling during HIV infection [34]. Within that response, interferon-linked proteins such as TRIM22, OAS1, and STAT2 increased while APOBEC3C/G/F/D, BST2, SERINC5, and CD4 decreased. Productively infected cells can therefore retain a strong antiviral-stress signature while losing selected proteins that directly constrain infection. This pattern is consistent with selective counter-restriction by lentiviral accessory proteins rather than global suppression of host defense [14,15,17–20].

TRIM22 provides one example of a restriction factor whose antiviral role includes transcriptional restriction [44], while broader ISG programs contain effectors with diverse mechanisms and potencies [45]. The immunometabolic modules connect this architecture to mechanism. SAMHD1 links restriction to intracellular deoxynucleotide availability and reverse transcription [14,46,47]. BST2 and SERINC5 act at membrane-dependent stages of release and virion infectivity [15,18–20], making lipid and cholesterol state biologically relevant rather than merely correlated. Autophagy, proteostasis, ER stress, and redox programs can further influence antiviral-protein turnover, antigen processing, inflammatory signaling, and infected-cell survival. These connections define experimentally testable edges between metabolism and intrinsic immunity.

CH25H nevertheless provides a biologically relevant example of how lipid metabolism can directly intersect antiviral restriction [48].

Previous genome-wide, proteomic, and single-cell studies established that HIV engages a broad host network [33–35,38–40] and that interferon effectors differ markedly in mechanism and antiviral potency [16]. The contribution here is to organize those observations across disease state, cell compartment, and protein polarity. This cross-layer view explains why RNA abundance alone cannot determine antiviral capacity and why apparently discordant datasets may be measuring different parts of the same system.

The same logic may extend to common comorbid states. Tuberculosis and diabetes alter myeloid activation, substrate use, redox balance, and inflammatory tone [30–32], each of which could shift the restrictome without changing the identity of its canonical factors. Testing these contexts would establish whether the architecture observed here is stable or remodeled across clinically important immune-metabolic environments.

This study has several limitations. The three datasets were generated in different cohorts, cell types, and experimental systems and were not subject matched. The cross-layer synthesis therefore cannot establish within-person molecular trajectories. Module scores depend on prespecified gene-set composition and dataset-specific feature coverage. Although participant-level analyses reduce pseudoreplication in the single-cell layer, the available annotations did not support a validated CD56bright/CD56dim NK-cell transcriptional comparison. The monocyte-subtype analysis demonstrates biologically meaningful composition effects but cannot reconstruct the exact cellular composition of the independent GSE157198 bulk samples. The sensing/death analyses measure expression rather than functional pathway activation. The proteomic analysis uses published source tables rather than raw-spectrum reprocessing, retains source missingness, and treats time-course abundance changes descriptively. Finally, all analyses remain observational; the mechanistic edges proposed in Fig 6 require experimental perturbation.

Fig 6 separates what is observed from what remains hypothetical. The observed layer consists of the GSE157198 clinical-state signal, participant-level cellular localization in GSE256089, and the PXD012263 protein-response patterns. These observations motivate, but do not prove, mechanistic relationships among interferon state, cell lineage, lipid/cholesterol metabolism, nucleotide availability, proteostasis/autophagy, redox biology, and restriction-factor abundance or function. The next step is therefore perturbational. Cholesterol synthesis or trafficking, nucleotide availability, redox state, autophagy, and proteostasis can be manipulated in defined immune-cell compartments while measuring both restriction-factor protein abundance and antiviral function. Such experiments would distinguish a correlated host state from a causal regulatory edge. Translationally, the present results argue that host-directed strategies should be evaluated in the cell type and molecular layer in which the relevant restriction program operates rather than assumed to exert uniform effects across immune compartments.

## Materials and methods

### Dataset selection and source-data processing

This study is an evidence-structured computational reanalysis of public, deidentified datasets and published source tables. The datasets were selected as complementary host-response evidence layers rather than as a subject-matched multi-omics cohort. GSE157198 contributed enriched monocyte RNA-sequencing profiles from healthy controls, ART-treated participants, ART-naive participants, and elite controllers [35]. GSE256089 contributed HTO-tagged PBMC single-cell/CITE-seq profiles from the ART time point of the A5354 cohort [33]. PXD012263 contributed published quantitative proteomic source data from HIV-exposed primary CD4 T cells [34]. Each dataset was analyzed within its own sampling and experimental structure; no cross-study subject matching or latent-factor integration was performed.

### Prespecified module framework and scoring

Before dataset-specific analysis, I defined eight modules: core HIV restriction factors; interferon/innate sensing; nucleotide metabolism; lipid/cholesterol membrane metabolism; mitochondrial/redox biology; glycolysis/PPP/inflammatory metabolism; autophagy/proteostasis/ER stress; and immune-cell state/activation/exhaustion. Membership was based on established restriction-factor, interferon, and immunometabolic biology [9–11,16,21,25,26] and was not tuned to any dataset. Module coverage was calculated separately for each molecular layer. Scores were computed from normalized abundance values using only detected genes or proteins, and all comparisons were interpreted in relation to the available feature coverage.

### Bulk monocyte RNA-sequencing analysis

For GSE157198, processed counts were parsed from the GEO supplementary workbook, mapped to gene symbols, and combined with curated sample metadata. Quality-control summaries included library size and detected-gene distributions. Module scores were calculated after coverage assessment, and prespecified contrasts compared untreated, ART-treated, elite-controller, and healthy-control groups. Benjamini-Hochberg adjustment controlled the false-discovery rate across module contrasts.

Within each module, gene-level contrasts were decomposed to identify the features contributing most strongly to group differences. Figs 2 and 3 were generated from the complete module- and gene-level contrast tables rather than from a post hoc subset selected for visualization.

### Bulk-monocyte robustness and conditional-coupling analyses

The primary GSE157198 core-restrictome disease effect was evaluated against three prespecified null schemes, each containing 1,000 random 37-gene sets, and with 37 leave-one-gene-out scores, each omitting one member of the 37-gene core module. Sample-level relationships between the core-restrictome, interferon/innate-sensing, and lipid/cholesterol scores were quantified using Spearman correlations. To test whether the lipid relationship was caused by direct gene-set overlap, the core-lipid analysis was repeated after removing CH25H from the lipid score. To test whether lipid coupling was independent of interferon, partial Spearman correlations were calculated conditioning first on the interferon/innate-sensing score and then on interferon plus clinical group. The permutation/null analyses, jackknife outputs, observed effect estimates, and conditional-correlation results were frozen before manuscript integration and were not recomputed for figure preparation.

### Single-cell analysis and reservoir metadata linkage

For GSE256089, 10x matrices were processed by sequencing lane, and gene-expression features were separated from antibody-derived and HTO features. Low-information cells were removed during preliminary quality control. Broad immune-cell identities were assigned using prespecified marker rules, and HTO labels were mapped to participant identifiers, recovering all 38 A5354 participants. Module scores were summarized at the cell, cell-type, participant, and participant-by-cell-type levels; participant-by-cell-type summaries were interpreted only when the cell-count thresholds documented in S5 Table were met.

Reservoir metadata were normalized before linkage. Participant identifiers were harmonized between module-score outputs and the published demographic and reservoir table. Participants were classified as undetectable, middle, or detectable according to the source categories. Module comparisons were performed at participant and participant-by-cell-type levels, with multiple-testing correction applied across the prespecified module tests.

### Participant-level NK specificity and monocyte-subtype analyses

For inferential single-cell analyses, the participant rather than the cell was the independent unit. For each of the 38 participants, broad-cell-type core-restrictome scores were summarized separately. NK enrichment was quantified as the difference between the participant’s NK score and that participant’s mean score across B cells, monocytes, and T cells. Specificity was evaluated against 1,000 random gene sets per prespecified matching scheme. Null sets were matched for the size of the core-restrictome module and for gene-expression/detection structure. Empirical upper-tail probabilities were calculated by comparing the observed participant-level NK enrichment with the matched null distributions. Existing single-cell monocyte annotations supported participant-paired CD14 and FCGR3A comparisons. Core-restrictome scores were therefore summarized for each subtype within each participant and analyzed as paired participant-level values. No validated participant-linked CD56bright/CD56dim single-cell annotation was available, so no NK-subtype transcriptional comparison was generated.

### Exploratory innate-sensing and cell-death targets

cGAS/MB21D1 and STING/TMEM173 were already represented in the prespecified interferon/innate-sensing framework. In response to peer review, IFI16, TREX1, CASP1, and CASP3 were added as explicitly exploratory targets without changing the membership or multiplicity structure of the original prespecified modules. Bulk-monocyte and participant-level single-cell expression of these targets was evaluated using the corresponding frozen dataset-specific analysis framework. These tests quantify transcript abundance and cell-context differences only. They were not interpreted as direct measurements of cGAS-STING activation, inflammasome activation, pyroptosis, caspase cleavage, or apoptosis.

### Proteomic overlay and cross-layer integration

For PXD012263, I used the published eLife source-data workbooks rather than reprocessing raw mass-spectrometry spectra. Candidate rows were matched to the curated framework by gene identifier. The historical published q < 0.05 WT-versus-mock selection was retained as the primary differential proteomic boundary. No quantitative protein-abundance imputation was performed in this reanalysis. Missing abundance entries in the source tables remained missing. Replicate summaries used available nonmissing values. A pseudocount of 1 was included in derived log2-ratio calculations and did not replace missing protein-abundance measurements. The identified fill operations were confined to non-abundance derived fields: a zero fallback was used for a derived protein-priority score and 99 was used for categorical sorting order. For sensitivity analysis, the existing published q values were evaluated at q < 0.01, q < 0.05, and q < 0.10 without recomputing the source-study multiple-testing adjustment. The historical q < 0.05 membership remained primary. Time-course abundance summaries were treated as descriptive source-table overlays and were not analyzed as new time-course differential-significance tests.

Cross-layer integration was evidence structured rather than based on a latent-factor model because the datasets differed in cohort, cell type, and experimental design. Evidence was indexed by dataset, molecular layer, biological context, module, direction, and interpretation. Convergence was defined as complementary support for the same organizational principle; no subject-level trajectory or causal relation was inferred across studies.

### Statistical analysis and reproducibility

All reported module contrasts were prespecified. Benjamini-Hochberg-adjusted P values were used for families of module comparisons, and both positive and null results were retained. Exact sample counts, feature coverage, effect estimates, P values, adjusted P values, cell-count thresholds, and source-to-figure mappings are provided in S1-S10 Tables. For the revision analyses, sample- or participant-level inference was used as appropriate to each dataset. Cells were not treated as independent replicates. Robustness analyses included permutation/null checks, leave-one-gene-out sensitivity, participant-paired comparisons, matched random-gene-set null distributions, partial correlations, monocyte-composition sensitivity, and FDR-threshold sensitivity using existing published q values. These analyses were used to test stability or specificity of existing claims rather than to redefine the prespecified biological modules. Author-generated analysis code and environment documentation underlying the reported findings are publicly available without restriction in Zenodo at https://doi.org/10.5281/zenodo.22396354.

### Ethics statement

This study used publicly available, deidentified processed datasets and published source-data tables. No new participants were recruited, and no new human specimens or animal experiments were generated. The original studies reported their own ethics approvals and consent procedures.

### Use of artificial intelligence tools

ChatGPT (OpenAI) was used to assist with code verification, manuscript organization, and language editing. Its outputs were checked against author-reviewed computational results, source-data documentation, and the cited literature. No primary data were generated, altered, or interpreted autonomously by the tool. The author reviewed and revised all affected code and text and is responsible for the final manuscript.

## Supporting information

S1 Table Dataset inventory and accession-level provenance. (XLSX)

S2 Table Curated restrictome-immunometabolism gene-set definitions. (XLSX)

S3 Table GSE157198 module scores and clinical-group contrasts. (XLSX)

S4 Table GSE157198 gene-level decomposition. (XLSX)

S5 Table GSE256089 cell-level and participant-level module summaries. (XLSX)

S6 Table GSE256089 reservoir-linked analyses. (XLSX)

S7 Table PXD012263 curated proteomic overlay. (XLSX)

S8 Table PXD012263 protein-polarity classifications. (XLSX)

S9 Table Cross-layer evidence synthesis. (XLSX)

S10 Table Figure-source and reproducibility register. (XLSX)

## Abbreviations

ART: antiretroviral therapy
CITE-seq: cellular indexing of transcriptomes and epitopes by sequencing
DC: dendritic cell
ER: endoplasmic reticulum
HTO: hashtag oligonucleotide
IFN: interferon
NK: natural killer
PBMC: peripheral blood mononuclear cell
PPP: pentose phosphate pathway
