# Supplementary material for: Cross-omics mapping of the human HIV restrictome across clinical states, immune-cell compartments, and protein responses
Source: PLoS One. 2026 Oct 13;21(10):e0356591. doi: 10.1371/journal.pone.0356591 (PMC13660893; doi:10.1371/journal.pone.0356591)
Supplement: Supplementary file 1 [file pone.0356591.s012.docx]

# Response to Reviewers

**Manuscript ID:** PONE-D-26-39263

**Revised title:** Cross-omics mapping of the human HIV restrictome across clinical states, immune-cell compartments, and protein responses

**Author:** Reuben S. Maghembe

Dear Dr. Carlos Manuel de Noronha and Reviewers,

Thank you for the careful and constructive evaluation of the manuscript. I have substantially revised the analytical interpretation and prepared additional robustness, cell-subtype, sensing/death-axis, and proteomic sensitivity analyses in response to the comments. I have also simplified the title and prose, clarified that the work is an evidence-structured computational reanalysis of independent public datasets rather than a subject-matched multi-omics study or a clinical meta-analysis, and revised the figures and their interpretation.

The principal scientific changes are: formal quantification of core-restrictome coupling with interferon and lipid/cholesterol programs; conditional analysis showing that the apparent lipid association is not independent of interferon; leave-one-gene-out and permutation/null robustness checks; participant-level matched random-gene-set analysis of NK-cell enrichment; participant-paired CD14 versus FCGR3A monocyte analysis and an independent bulk-monocyte composition sensitivity analysis; exploratory analysis of cGAS/STING and reviewer-requested sensing/death genes; clarification of missing-value handling in PXD012263; and sensitivity analysis across q<0.01, q<0.05, and q<0.10. All inferential single-cell comparisons use participants rather than cells as independent units.

I respond point by point below.

## Journal requirements

### J01. PLOS ONE style requirements and file naming

**Response**

I have prepared the revision for PLOS ONE formatting rather than the earlier transfer format. The revised title is in sentence case and has been simplified to:

**Cross-omics mapping of the human HIV restrictome across clinical states, immune-cell compartments, and protein responses**

The short title is:

**Mapping the human HIV restrictome**

Figure citations and legends use the PLOS form “Fig 1,” “Fig 2,” and so forth. The final submission package will include the clean revised manuscript, a marked-up version showing changes, the Response to Reviewers, the six separately uploaded frozen main figures, and the required supporting-information files.

Zotero-preserving manuscript integration is complete. Final submission file names will be checked during final upload-set assembly; this response uses stable section, figure, and supporting-information identifiers rather than provisional line numbers.

### J02. Data availability and repository deposition

**Response**

The manuscript already identifies the three public source datasets using their permanent accessions: GSE157198, GSE256089, and PXD012263. The processed module scores, gene-level contrasts, participant-level single-cell summaries, reservoir-linked analyses, proteomic overlays, evidence tables, and figure-source registers are supplied in the supporting information.

The revised manuscript now identifies the permanent Zenodo repository for author-generated analysis code and derived reproducibility materials: https://doi.org/10.5281/zenodo.22396354.

### J03. Amended Funding Statement and Role of Funders

**Response**

I confirm that this study received no specific funding. My commercial affiliation with AfroBiomics Co. Ltd. is declared, but AfroBiomics Co. Ltd. provided no salary support for this study and no study-specific financial, material, computing, software, administrative, publication, or other support. AfroBiomics Co. Ltd. had no role in study design, data collection, analysis, interpretation, decision to publish, or manuscript preparation.

**Funding Statement**

The author received no specific funding for this work. Reuben S. Maghembe is affiliated with AfroBiomics Co. Ltd.; however, AfroBiomics Co. Ltd. provided no salary, financial, research-material, computing, software, administrative, publication, or other material support for this study and had no role in study design, data collection and analysis, interpretation, decision to publish, or preparation of the manuscript.

### J04. Updated Competing Interests Statement

**Response**

I confirm my commercial affiliation as Chief Executive Officer of AfroBiomics Co. Ltd. AfroBiomics Co. Ltd. provided no financial or material support for this study and had no role in study design, data collection, analysis, interpretation, decision to publish, or manuscript preparation. I have no other competing interests to declare, and AfroBiomics Co. Ltd. imposes no restrictions on sharing the study's data, code, materials, or publication.

**Competing Interests Statement**

I am Chief Executive Officer of AfroBiomics Co. Ltd. I have no other competing interests to declare. This commercial affiliation provided no financial or material support for this study and had no role in study design, data collection, analysis, interpretation, decision to publish, or manuscript preparation. AfroBiomics Co. Ltd. imposes no restrictions on sharing the study's data, code, materials, or publication. This does not alter my adherence to PLOS ONE policies on sharing data and materials.

### J05. Reviewer-recommended references

**Response**

I evaluated the four publications specifically recommended by Reviewer 2. They are relevant to the revised discussion of CH25H-mediated restriction, TRIM22-mediated restriction, dendritic-cell antiviral programs, and interferon-stimulated effector networks. They were incorporated through the protected Zotero-preserving reference-integration workflow at the relevant manuscript locations.

The requested publications are identified by PMIDs 23273844, 21345949, 31244850, and 31283436. The bibliographic record associated with PMID 21345949 also has a later correction record, PMID 39868830, which was handled during reference integration as bibliographic provenance without treating it as an additional reviewer-requested citation.

# Reviewer 1

## R1.01. Innate sensing and cell-death axis

**Comment addressed**

The reviewer noted that the original framework did not adequately expose the HIV-relevant sensing and cell-death axis, including IFI16, STING, TREX1, caspase-1/inflammasome biology, caspase-3, and the absence of cGAS from the Results.

**Response**

I agree that this axis required more explicit treatment. cGAS and STING were not actually absent from the prespecified framework: cGAS/MB21D1 and STING/TMEM173 were already represented in the interferon/innate-sensing module. I nevertheless added a focused analysis that makes these genes explicit and added IFI16, TREX1, CASP1, and CASP3 as clearly labeled reviewer-requested exploratory targets rather than retrospectively redefining the prespecified modules.

In GSE157198, TREX1 showed clear FDR-supported variation, whereas the bulk cGAS and STING contrasts were not FDR-supported. In GSE256089, IFI16, STING/TMEM173, and CASP3 were higher in NK cells, whereas TREX1 and CASP1 were higher in monocytes; cGAS/CGAS did not show a material NK-versus-monocyte difference.

I have explicitly bounded the interpretation. Transcript abundance does not demonstrate cGAS-STING pathway activation; CASP1 expression does not demonstrate inflammasome activation or pyroptosis; and CASP3 expression does not demonstrate executioner-caspase activation or apoptosis. The revised manuscript therefore presents these findings as expression-state observations and mechanistic hypotheses rather than functional cell-death measurements.

## R1.02. Formal statistics for the coupling claims

**Comment addressed**

The reviewer requested quantitative statistical support for the core-restrictome coupling claims.

**Response**

I added formal sample-level coupling statistics in GSE157198. Core-restrictome and interferon/innate-sensing scores were strongly correlated (Spearman r=0.799718, p=7.46724×10^-11). Core-restrictome and lipid/cholesterol scores were also correlated in the unadjusted analysis (Spearman r=0.451163, p=0.00211408).

These values are now stated directly in the Results and Fig 2 legend rather than describing coupling qualitatively.

## R1.03. Whether lipid coupling is independent of interferon

**Comment addressed**

The reviewer asked whether the core-restrictome/lipid association persists independently of the shared interferon axis.

**Response**

I performed two sensitivity analyses. First, removing CH25H, which overlaps the core and lipid/cholesterol modules, still gave an unadjusted association (Spearman r=0.424242, p=0.00410444). However, the association disappeared when interferon was controlled: partial Spearman r=0.059104, p=0.703132 after conditioning on IFN, and r=-0.017597, p=0.909734 after conditioning on both IFN and clinical group.

I therefore revised the interpretation throughout the manuscript. Lipid/cholesterol activity is no longer described as an independently coupled restrictome program. Instead, the data support a **shared IFN-associated immunometabolic architecture** in this cohort.

## R1.04. NK-cell lineage baseline and specificity

**Comment addressed**

The reviewer noted that the original observation that NK cells had the highest score could reflect constitutive lineage-specific expression rather than specific restrictome enrichment.

**Response**

I agree that simple rank ordering was insufficient. I therefore moved the analysis to the participant level and compared each participant's NK core-restrictome score with that participant's mean score across B cells, monocytes, and T cells. Across 38 participants, the mean NK-minus-reference difference was 0.105124, with dz=3.8490 and paired p=5.551754×10^-24. NK cells had the highest score in 35 of 38 participants.

More importantly, I compared the observed effect with 1,000 random gene sets per prespecified matching scheme, controlling for gene-set size and expression/detection structure. The NK-versus-reference enrichment remained unusual under these matched nulls, with empirical upper-tail p values of 0.001998, 0.007992, and 0.000999 across the frozen matching schemes.

I have therefore changed the interpretation. The revised manuscript describes the NK pattern as constitutive/developmental antiviral readiness together with specific enrichment of the curated restrictome beyond matched background gene sets. It does **not** interpret the NK signal as uniform HIV-induced activation.

All inferential comparisons use participants, not cells, as independent units.

## R1.05. Monocyte subset composition

**Comment addressed**

The reviewer requested evaluation of whether the bulk-monocyte clinical-state result could be driven by monocyte subset composition.

**Response**

I addressed this in two complementary ways.

First, in GSE256089, I compared participant-paired CD14 and FCGR3A monocyte subtypes. Across 38 participants, there were 37,081 CD14 cells and 7,581 FCGR3A cells. The core-restrictome score was higher in FCGR3A than CD14 monocytes by 0.157650 on average (dz=2.95165; paired p=4.773127×10^-20), with FCGR3A exceeding CD14 in 37 of 38 participants. Monocyte composition was therefore biologically associated with restrictome state.

Second, I tested three transcriptional proxies of CD14-FCGR3A balance in the independent GSE157198 bulk-monocyte cohort. Adjustment for these proxies left the ART-naive clinical-state effect essentially unchanged at approximately 1.14–1.17, with p values approximately 0.0006–0.0011.

The revised interpretation is:

**Monocyte subtype composition is biologically associated with restrictome activity, but transcriptional proxies of CD14-FCGR3A balance did not account for the clinical-state-associated restrictome elevation observed in the independent GSE157198 bulk-monocyte cohort.**

## R1.06. Mechanistic and translational interpretation of Fig 6

**Comment addressed**

The reviewer considered the architecture useful but requested an explicit mechanistic hypothesis and translational interpretation.

**Response**

I revised Fig 6 around three deliberately separated levels:

1. observed evidence;

2. noncausal mechanistic hypotheses; and

3. perturbational tests.

The figure no longer presents arrows as established causal pathways. Instead, the observed transcriptomic, cell-localization, and proteomic findings motivate testable hypotheses concerning IFN state, immune-cell lineage, lipid/cholesterol state, nucleotide availability, proteostasis/autophagy, and redox context.

The revised Discussion specifies perturbational tests such as manipulating cholesterol synthesis, nucleotide availability, redox state, or autophagy/proteostasis while measuring both restriction-factor abundance and antiviral function in defined immune-cell compartments. The translational implication is correspondingly bounded: host-directed interventions should be evaluated in the cell type and molecular layer in which the relevant restriction program operates rather than assumed to act uniformly across immune compartments.

## R1.07. Title and prose complexity

**Comment addressed**

The reviewer recommended a simpler title and clearer wording.

**Response**

I agree and have substantially simplified the title and manuscript-wide prose.

The revised title is:

**Cross-omics mapping of the human HIV restrictome across clinical states, immune-cell compartments, and protein responses**

I also replaced several abstract and Introduction formulations that could imply a stronger integrated or causal model than the data support. The revised framing states that the data suggest context-specific combinations of antiviral defenses across clinical states and immune-cell compartments rather than a uniform intrinsic antiviral program.

# Reviewer 2

## R2.01. Random-gene-set / permutation robustness

**Comment addressed**

The reviewer requested null testing to establish that both the monocyte disease-state separation and NK-cell enrichment were not generic transcriptomic background effects.

**Response**

I addressed the two parts separately because they involve different experimental structures.

### Bulk monocyte disease-state signal

The GSE157198 robustness analysis used three prespecified null schemes, with 1,000 random 37-gene sets per scheme (3,000 total). The observed ART-naive-minus-healthy core-restrictome difference was 1.153725 (Welch p=0.000745751) and was more extreme than the null distribution in all three schemes (empirical p=0.000999001 in each; BH-adjusted empirical p=0.002997 in each).

The observed core-IFN correlation (Spearman r=0.799718) was also highly unusual under the null schemes, with empirical p values of 0.000999001, 0.001998002, and 0.000999001 and corresponding BH-adjusted values of 0.002997, 0.004995, and 0.002997. By contrast, the unadjusted core-lipid relationship was less stable under expression-matched nulls (empirical p=0.0239760, 0.0989011, and 0.0929071), and the IFN-adjusted core-lipid association remained null under all schemes (empirical p=0.709291, 0.700300, and 0.715285). These results support the specificity and robustness of the disease-state and core-IFN signals while reinforcing the revised conclusion that the lipid association is not independent of interferon.

### NK-cell enrichment

For the NK analysis, I generated 1,000 size- and expression/detection-matched random gene sets per prespecified null scheme. The observed participant-level NK enrichment exceeded the matched background distributions, with frozen empirical upper-tail p values of 0.001998, 0.007992, and 0.000999 across the relevant matching schemes.

Thus, the revised manuscript no longer relies on the observation that NK cells simply rank highest.

## R2.02. Leave-one-gene-out analysis of the 37-gene core restrictome

**Comment addressed**

The reviewer requested a jackknife analysis to determine whether one dominant gene drives the GSE157198 clinical-state effect.

**Response**

I performed 37 leave-one-core-gene-out analyses. The ART-naive-versus-healthy disease effect retained the same direction in **37/37** jackknife analyses. This supports a distributed module-level signal rather than an effect dependent on one dominant member of the core restrictome.

The result is now stated in the Results and Fig 2 legend.

## R2.03. Higher-resolution NK and monocyte subtypes

**Comment addressed**

The reviewer suggested CD56dim versus CD56bright NK cells and CD14-positive versus CD16-positive monocytes where the data support such analysis.

**Response**

I performed the higher-resolution analysis only where valid labels existed.

For monocytes, the historical single-cell annotations supported CD14 and FCGR3A subtypes, enabling participant-paired analysis. FCGR3A core-restrictome scores exceeded CD14 scores in 37 of 38 participants, with a mean difference of 0.157650 and paired p=4.773127×10^-20.

For NK cells, the single-cell dataset did **not** contain validated participant-linked CD56bright and CD56dim single-cell labels. Although separate flow-cytometry metadata contained CD56dim information, it did not establish matched single-cell CD56bright/CD56dim assignments suitable for the requested transcriptomic comparison. I therefore did not manufacture an NK-subtype classification from marker assumptions that were not validated in the source dataset.

The limitation is now stated explicitly.

## R2.04. Missing-value imputation in PXD012263

**Comment addressed**

The reviewer requested clarification of missing-value handling.

**Response**

I traced the complete time-course overlay and polarity workflow to the source tables and adjudicated every imputation-related operation. No quantitative protein-abundance imputation was identified in this reanalysis. Missing source-table abundance values were retained as missing.

Replicate summaries used available nonmissing source values, and a pseudocount of 1 was included in derived log2-ratio calculations. The pseudocount did not replace missing protein-abundance measurements and is therefore distinct from missing-value imputation. The identified fill operations were likewise non-abundance operations: fillna(0) was used for a derived protein-priority score, and fillna(99) was used for categorical sorting order.

I have revised the Methods to state this explicitly.

## R2.05. FDR-threshold sensitivity

**Comment addressed**

The reviewer requested evaluation beyond q<0.05.

**Response**

I evaluated the existing published q values at q<0.01, q<0.05, and q<0.10. Among 137 q-evaluable curated genes, the thresholds selected 10, 19, and 25 genes, respectively. The q<0.05 analysis reproduced the historical 19-gene membership exactly.

Mixed protein-response polarity persisted across the thresholds, although the relative balance of increased, depleted, and stable/subtle proteins varied with threshold. I therefore retain historical q<0.05 as the **primary** proteomic analysis and present q<0.01 and q<0.10 strictly as sensitivity analyses.

The time-course abundance panels remain descriptive and are not presented as independent differential-significance tests.

## R2.06. Terminology and study type

**Comment addressed**

The reviewer recommended distinguishing the study from a clinical PRISMA meta-analysis.

**Response**

I agree. The manuscript now consistently uses the term:

**“evidence-structured computational reanalysis of public datasets”**

The three datasets are described as complementary evidence layers. They are not described as a subject-matched multi-omics cohort, and the manuscript does not imply within-subject cross-platform trajectories.

## R2.07. Constitutive antiviral priming of NK cells

**Comment addressed**

The reviewer asked that the Discussion acknowledge the constitutive antiviral readiness of NK cells.

**Response**

I added this distinction to the revised Discussion. The high NK core-restrictome score is interpreted as a combination of lineage/developmental antiviral readiness and specific curated-restrictome enrichment beyond size- and expression/detection-matched null gene sets.

I explicitly avoid interpreting the broad NK signal as evidence that HIV uniformly induced the program in NK cells.

## R2.08. Recommended references

**Comment addressed**

The reviewer recommended four publications concerning CH25H, TRIM22, dendritic-cell programs, and ISG effector networks.

**Response**

I evaluated all four and consider them relevant to the revised biological interpretation. They were incorporated through the protected Zotero-preserving reference-integration workflow at the relevant manuscript locations:

- PMID 23273844 — CH25H and viral-entry restriction;

- PMID 21345949 — TRIM22-mediated HIV transcriptional restriction;

- PMID 31244850 — dendritic-cell antiviral programs;

- PMID 31283436 — interferon-stimulated effector networks.

The later correction record PMID 39868830 was handled as bibliographic provenance for PMID 21345949 rather than as a fifth reviewer-requested reference.

Final reference numbering was generated through Zotero during the protected reference-integration stage.

## R2.09. Textual and editorial revisions

**Response**

I simplified the title, Abstract, Introduction conclusion, Results interpretation, Discussion, and figure legends. I also standardized the study description as an evidence-structured computational reanalysis and maintained single-author wording throughout.

The revised text avoids claims of subject-matched multi-omics, avoids causal language unsupported by the datasets, and distinguishes observed expression from pathway activation and cell-death mechanisms.

## R2.11. Figure cleanup and accessibility

**Comment addressed**

The reviewer requested removal of working notes, correction of text collisions, improved box centering, consistent typography, and consideration of color accessibility.

**Response**

I addressed these presentation issues in the final frozen figure set. The revised versions remove the working annotations, resolve the identified collisions, improve text placement and typography, and retain the scientifically approved panel architecture.

No scientific model was refitted and no figure-level statistical analysis was rerun merely for presentation cleanup.

## Closing response

I appreciate the reviewers' suggestions. The additional analyses materially strengthened the manuscript by distinguishing robust clinical-state and NK signals from background structure, separating lipid/cholesterol association from the shared interferon axis, identifying a monocyte-subtype contribution without attributing the independent bulk disease effect to composition, and more clearly bounding the mechanistic interpretation of the sensing/death and proteomic results.

The revised manuscript now presents the human HIV restrictome as a context-dependent pattern observed across independent public evidence layers, with explicit separation between observed associations, mechanistic hypotheses, and future perturbational tests.

Sincerely,

Reuben S. Maghembe
