# Supplementary material for: Cross-omics mapping of the human HIV restrictome across clinical states, immune-cell compartments, and protein responses
Source: PLoS One. 2026 Oct 13;21(10):e0356591. doi: 10.1371/journal.pone.0356591 (PMC13660893; doi:10.1371/journal.pone.0356591)
Supplement: Peer review history

# Author comment: Author response to Decision Letter 0

**DOI:** 10.1371/journal.pone.0356591.r001

**Transfer Alert**

This paper was transferred from another journal. As a result, its full editorial history (including decision letters, peer reviews and author responses) may not be present.

5 Aug 2026

# Peer review: Decision Letter 0

**DOI:** 10.1371/journal.pone.0356591.r002

**Authors:** Carlos de Noronha

1 Sep 2026

PONE-D-26-39263Immune-cell compartmentalization, interferon-immunometabolic coupling, and proteomic polarity define the human HIV restrictomePLOS One

Dear Dr. Maghembe,

Thank you for submitting your manuscript to PLOS One. After careful consideration, we feel that it has merit but does not yet fully meet PLOS One’s publication criteria as it currently stands. Therefore, we invite you to submit a revised version of the manuscript that addresses the points raised during the review process.

Please submit your revised manuscript by Oct 31 2026 11:59PM. If you will need more time than this to complete your revisions, please reply to this message or contact the journal office at plosone@plos.org. When you're ready to submit your revision, log on to https://www.editorialmanager.com/pone/ and select the 'Submissions Needing Revision' folder to locate your manuscript file.

Please include the following items when submitting your revised manuscript:

- A letter that responds to each point raised by the academic editor and reviewer(s). You should upload this letter as a separate file labeled 'Response to Reviewers'.
- A marked-up copy of your manuscript that highlights changes made to the original version. You should upload this as a separate file labeled 'Revised Manuscript with Track Changes'.
- An unmarked version of your revised paper without tracked changes. You should upload this as a separate file labeled 'Manuscript'.

If you would like to make changes to your financial disclosure, please include your updated statement in your cover letter. Guidelines for resubmitting your figure files are available below the reviewer comments at the end of this letter.

If applicable, we recommend that you deposit your laboratory protocols in protocols.io to enhance the reproducibility of your results. Protocols.io assigns your protocol its own identifier (DOI) so that it can be cited independently in the future. For instructions see: https://journals.plos.org/plosone/s/submission-guidelines#loc-laboratory-protocols. Additionally, PLOS One offers an option for publishing peer-reviewed Lab Protocol articles, which describe protocols hosted on protocols.io. Read more information on sharing protocols at https://plos.org/protocols?utm_medium=editorial-email&utm_source=authorletters&utm_campaign=protocols.

As the corresponding author, your ORCID iD is verified in the submission system and will appear in the published article. PLOS supports the use of ORCID, and we encourage all coauthors to register for an ORCID iD and use it as well. Please encourage your coauthors to verify their ORCID iD within the submission system before final acceptance, as unverified ORCID iDs will not appear in the published article. *Only*  the individual author can complete the verification step; PLOS staff *cannot*  verify ORCID iDs on behalf of authors.

We look forward to receiving your revised manuscript.

Kind regards,

Carlos Manuel de Noronha, Sc.D.

Academic Editor

PLOS One

Journal Requirements:

When submitting your revision, we need you to address these additional requirements.

1. Please ensure that your manuscript meets PLOS ONE's style requirements, including those for file naming. The PLOS ONE style templates can be found at

https://journals.plos.org/plosone/s/file?id=wjVg/PLOSOne_formatting_sample_main_body.pdf and

https://journals.plos.org/plosone/s/file?id=ba62/PLOSOne_formatting_sample_title_authors_affiliations.pdf

2. In the online submission form, you indicated that your data will be submitted to a repository upon acceptance.  We strongly recommend all authors deposit their data before acceptance, as the process can be lengthy and hold up publication timelines. Please note that, though access restrictions are acceptable now, your entire minimal  dataset will need to be made freely accessible if your manuscript is accepted for publication. This policy applies to all data except where public deposition would breach compliance with the protocol approved by your research ethics board. If you are unable to adhere to our open data policy, please kindly revise your statement to explain your reasoning and we will seek the editor's input on an exemption.

3. Thank you for stating the following in the Competing Interests section:

“The author has declared that no competing interests exist.”

We note that one or more of the authors are employed by a commercial company: “Department of Omics and Computational Biology, AfroBiomics Co. Ltd.”

1. Please provide an amended Funding Statement declaring this commercial affiliation, as well as a statement regarding the Role of Funders in your study. If the funding organization did not play a role in the study design, data collection and analysis, decision to publish, or preparation of the manuscript and only provided financial support in the form of authors' salaries and/or research materials, please review your statements relating to the author contributions, and ensure you have specifically and accurately indicated the role(s) that these authors had in your study. You can update author roles in the Author Contributions section of the online submission form.

Please also include the following statement within your amended Funding Statement.

“The funder provided support in the form of salaries for authors [insert relevant initials], but did not have any additional role in the study design, data collection and analysis, decision to publish, or preparation of the manuscript. The specific roles of these authors are articulated in the ‘author contributions’ section.”

If your commercial affiliation did play a role in your study, please state and explain this role within your updated Funding Statement.

2. Please also provide an updated Competing Interests Statement declaring this commercial affiliation along with any other relevant declarations relating to employment, consultancy, patents, products in development, or marketed products, etc.

Within your Competing Interests Statement, please confirm that this commercial affiliation does not alter your adherence to all PLOS ONE policies on sharing data and materials by including the following statement: "This does not alter our adherence to  PLOS ONE policies on sharing data and materials.” (as detailed online in our guide for authors http://journals.plos.org/plosone/s/competing-interests) . If this adherence statement is not accurate and  there are restrictions on sharing of data and/or materials, please state these. Please note that we cannot proceed with consideration of your article until this information has been declared.

Please include both an updated Funding Statement and Competing Interests Statement in your cover letter. We will change the online submission form on your behalf.

4. If the reviewer comments include a recommendation to cite specific previously published works, please review and evaluate these publications to determine whether they are relevant and should be cited. There is no requirement to cite these works unless the editor has indicated otherwise.

[Note: HTML markup is below. Please do not edit.]

Reviewers' comments:

Reviewer's Responses to Questions

**Comments to the Author**

1. Is the manuscript technically sound, and do the data support the conclusions?

The manuscript must describe a technically sound piece of scientific research with data that supports the conclusions. Experiments must have been conducted rigorously, with appropriate controls, replication, and sample sizes. The conclusions must be drawn appropriately based on the data presented.

Reviewer #1: Partly

Reviewer #2: Yes

**********

2. Has the statistical analysis been performed appropriately and rigorously?

Reviewer #1: I Don't Know

Reviewer #2: Yes

**********

3. Have the authors made all data underlying the findings in their manuscript fully available?

The PLOS Data policy requires authors to make all data underlying the findings described in their manuscript fully available without restriction, with rare exception (please refer to the Data Availability Statement in the manuscript PDF file). The data should be provided as part of the manuscript or its supporting information, or deposited to a public repository. For example, in addition to summary statistics, the data points behind means, medians and variance measures should be available. If there are restrictions on publicly sharing data—e.g. participant privacy or use of data from a third party—those must be specified.

Reviewer #1: Yes

Reviewer #2: Yes

**********

4. Is the manuscript presented in an intelligible fashion and written in standard English?

PLOS ONE does not copyedit accepted manuscripts, so the language in submitted articles must be clear, correct, and unambiguous. Any typographical or grammatical errors should be corrected at revision, so please note any specific errors here.

Reviewer #1: Yes

Reviewer #2: Yes

**********

5. Review Comments to the Author

Please use the space provided to explain your answers to the questions above. You may also include additional comments for the author, including concerns about dual publication, research ethics, or publication ethics. (Please upload your review as an attachment if it exceeds 20,000 characters)

Reviewer #1: This single-author study reanalyzes three public datasets (bulk monocyte RNA-seq GSE157198, PBMC single-cell/CITE-seq GSE256089, and primary CD4 T-cell proteomics PXD012263) through a prespecified eight-module framework linking HIV restriction factors to interferon and immunometabolic programs. The main claims are that untreated infection produces the strongest interferon-coupled monocyte restrictome while elite controllers do not, that restriction and interferon/lipid programs localize to different immune compartments (NK versus monocyte and DC), and that infected CD4 T cells show bidirectional protein-level remodeling, with APOBEC3C/D/F/G, BST2, SERINC5 and CD4 depleted while TRIM22, OAS1 and STAT2 are induced.

In conclusion, the author suggests that the restrictome is a compartmentalized cell-state-dependent program rather than a uniform ISG set. This conclusion is reasonable and is the main contribution presented in this work.

The organizing question is worthwhile and the cross-layer framing may have contribution to the field: it offers a potential explanation for why transcript abundance, cell-type localization and protein behavior diverge in chronic infection. The prespecified module design, retention of null results, and explicit limitations section are commendable. However, the work is currently all descriptive and the analysis does not yet support the strength of the conclusions. Three issues are material. First, the curated framework omits the innate sensing and cell-death axis most relevant to HIV pathogenesis and CD4 T cell depletion, including IFI16, STING1 and TREX1 sensing of reverse-transcribed products and the caspase-1/inflammasome versus caspase-3 death pathways that govern CD4 depletion; CGAS is listed but never appears in any result. Second, the central coupling claims lack supporting statistics, the lipid-restrictome association is not separated from the shared interferon axis, and the headline NK finding is not controlled for lineage-driven baseline expression or for monocyte subset composition in the bulk layer. Third, Figure 6A is helpful for understanding how the HIV restrictome is organized by cell identity and immunometabolic programs. However it presents an architecture without a mechanistic hypothesis or translational interpretation, which leaves the paper's most interesting result underexploited.

A general note, the article language in many parts (starting from the title) is unnecessarily complex and difficult to interpret. To avoid losing readers from the outset, I would recommend a simpler, clearer, and more elegant title.

For example:

HIV Restrictome Organizeation by Clinical State, Cell Identity, and Immunometabolic Programs

The HIV Restrictome Across Immune Cells and Clinical States

Mapping the HIV Restrictome Across Immune Cells

Similarly, the conclusion, as stated in the introduction uses un-necessary complex language: "Together, the results support a cross-layer model in 5 which intrinsic antiviral capacity emerges from cell-state-specific network organization rather than uniform induction of a fixed gene set".

A more simplified conclusion could be: The data suggest that cells employ cell-specific combinations of antiviral defense mechanisms rather than a uniform intrinsic antiviral response.

Reviewer #2: In this manuscript, the author presents a computational synthesis investigating whether the human HIV restrictome functions as a static list of antiviral effectors or an integrated network coupled to IFN signaling and immunometabolism. The author applied an eight-module framework across three public datasets: bulk RNA sequencing of monocytes across clinical states (GSE157198), single cell CITE seq profiling of PBMCs linked to viral reservoir categories (GSE256089), and time resolved CD4+ T cell quantitative proteomics (PXD012263). Key findings show that untreated HIV drives the highest core restriction factor activity and tightest coregulation with IFN signaling in monocytes, whereas elite controllers lack a baseline hyper restrictome state. Single cell profiling localizes core restriction activity primarily to NK cells, while IFN signaling and lipid/cholesterol metabolism concentrate in monocytes and DCs. Furthermore, broad module scores do not separate patient viral reservoir categories after Benjamini Hochberg false discovery rate (FDR) correction. Finally, infected CD4+ T cells display a bidirectional proteomic response where canonical targets (i.e., APOBEC3 family, BST2, etc.) are depleted by viral accessory proteins while unantagonized ISGs are strongly upregulated.

This manuscript aligns with PLoS ONE publication criteria, with emphasis on technical soundness, methodological rigor, open data, and proper interpretation. The analytical design utilizes prespecified biological modules to eliminate post hoc selection bias, applies appropriate Benjamini Hochberg FDR adjustments across module contrasts, and avoids overclaiming causality or within person trajectories across non-subject matched cohorts. Retaining null results, such as the lack of reservoir category separation after FDR adjustment, demonstrates scientific integrity. Additionally, relying on public repositories and committing to open code sharing satisfies journal reproducibility requirements.

The study possesses several strengths, most notably its multi layered resolution that captures post transcriptional viral degradation (i.e., Vif, Vpu, or Nef targets) undetectable by transcriptomics alone. Again, predefining the eight biological modules prevents post hoc bias when analyzing complex datasets. Resolving cell lineage division of labor (NK effector profiles versus myeloid metabolic and IFN signaling) helps clarify why bulk transcriptomic studies often yield discordant results. Finally, Instead of using complex math to force together data from completely different groups of people, the study neatly organizes the findings into a clear, side-by-side comparison table. This keeps the analysis scientifically honest and accurate.

The primary weaknesses stem from structural constraints inherent to reanalyzing public data, most of which are commendably acknowledged by the author in the Discussion. Because the datasets derive from separate cohorts and experimental systems, within-subject longitudinal trajectories and direct causal relationships cannot be established. As noted in the manuscript, mass spectrometry coverage limitations create scoring depth imbalances relative to bulk RNA sequencing, and the single-cell CITE-seq analysis relies on broad marker rules rather than fine subpopulation resolution. Lastly, the author appropriately bounds the study as observational and hypothesis-generating, transparently highlighting the absence of wet-lab functional perturbation assays to validate predicted immunometabolic interactions.

To strengthen technical soundness and ensure result stability, several computational and textual revisions are recommended. First, the author should perform permutation testing against 1,000 random gene sets of equivalent size to confirm that monocyte disease state separation and NK cell restriction enrichment reflect specific biological pathways rather than background transcriptomic noise. A ‘jackknife’ or ‘leave one out sensitivity’ analysis on the 37 gene core restrictome module should also be conducted to verify that monocyte contrasts are not driven by single dominant genes such as ISG15 or IFITM1. Where cell counts permit, single cell scores should be evaluated at higher resolution, comparing CD56 dim versus CD56 bright NK cells and CD14 positive versus CD16 positive monocytes. For dataset PXD012263, missing value imputation procedures should be clarified, and sensitivity across alternative FDR thresholds beyond q < 0.05 should be confirmed.

Textually, the manuscript should consistently define the work as an evidence structured computational reanalysis to avoid confusion with clinical PRISMA meta-analyses. The Discussion should be updated to note NK cells' constitutive antiviral priming. Four key references should be added to reinforce biological mechanisms: Liu et al. 2013 (PMID 23273844) for CH25H entry restriction, Kajaste-Rudnitski et al. 2011 (PMID 21345949) for TRIM22 transcriptional restriction, Martin-Gayo & Yu 2019 (PMID 31244850) for dendritic cell programs, and Schoggins 2019 (PMID 31283436) for ISG effector networks.

Finally, to prepare the figures for final publication, remove leftover working draft notes in Figures 2F and 6B. Eliminate text collisions and overlaps of Figures 2C, 4B, 4D, 5D and 6B. Center text within boxes in Figures 1. Ensure consistent font sizing and consider colorblind-safe color schemes.

**********

6. PLOS authors have the option to publish the peer review history of their article (what does this mean?). If published, this will include your full peer review and any attached files.

If you choose “no”, your identity will remain anonymous but your review may still be made public.

**Do you want your identity to be public for this peer review?**  For information about this choice, including consent withdrawal, please see our Privacy Policy.

Reviewer #1: No

Reviewer #2: No

**********

[NOTE: If reviewer comments were submitted as an attachment file, they will be attached to this email and accessible via the submission site. Please log into your account, locate the manuscript record, and check for the action link "View Attachments". If this link does not appear, there are no attachment files.]

To ensure your figures meet our technical requirements, please review our figure guidelines: https://journals.plos.org/plosone/s/figures

You may also use PLOS’s free figure tool, NAAS, to help you prepare publication quality figures: https://journals.plos.org/plosone/s/figures#loc-tools-for-figure-preparation.

NAAS will assess whether your figures meet our technical requirements by comparing each figure against our figure specifications.

# Author comment: Author response to Decision Letter 1

**DOI:** 10.1371/journal.pone.0356591.r003

15 Sep 2026

Response to Reviewers

Manuscript ID: PONE-D-26-39263

Revised title: Cross-omics mapping of the human HIV restrictome across clinical states, immune-cell compartments, and protein responses

Author: Reuben S. Maghembe

Dear Dr. Carlos Manuel de Noronha and Reviewers,

Thank you for the careful and constructive evaluation of the manuscript. I have substantially revised the analytical interpretation and prepared additional robustness, cell-subtype, sensing/death-axis, and proteomic sensitivity analyses in response to the comments. I have also simplified the title and prose, clarified that the work is an evidence-structured computational reanalysis of independent public datasets rather than a subject-matched multi-omics study or a clinical meta-analysis, and revised the figures and their interpretation.

The principal scientific changes are: formal quantification of core-restrictome coupling with interferon and lipid/cholesterol programs; conditional analysis showing that the apparent lipid association is not independent of interferon; leave-one-gene-out and permutation/null robustness checks; participant-level matched random-gene-set analysis of NK-cell enrichment; participant-paired CD14 versus FCGR3A monocyte analysis and an independent bulk-monocyte composition sensitivity analysis; exploratory analysis of cGAS/STING and reviewer-requested sensing/death genes; clarification of missing-value handling in PXD012263; and sensitivity analysis across q<0.01, q<0.05, and q<0.10. All inferential single-cell comparisons use participants rather than cells as independent units.

I respond point by point below.

Journal requirements

J01. PLOS ONE style requirements and file naming

Response

I have prepared the revision for PLOS ONE formatting rather than the earlier transfer format. The revised title is in sentence case and has been simplified to:

Cross-omics mapping of the human HIV restrictome across clinical states, immune-cell compartments, and protein responses

The short title is:

Mapping the human HIV restrictome

Figure citations and legends use the PLOS form “Fig 1,” “Fig 2,” and so forth. The final submission package will include the clean revised manuscript, a marked-up version showing changes, the Response to Reviewers, the six separately uploaded frozen main figures, and the required supporting-information files.

Zotero-preserving manuscript integration is complete. Final submission file names will be checked during final upload-set assembly; this response uses stable section, figure, and supporting-information identifiers rather than provisional line numbers.

J02. Data availability and repository deposition

Response

The manuscript already identifies the three public source datasets using their permanent accessions: GSE157198, GSE256089, and PXD012263. The processed module scores, gene-level contrasts, participant-level single-cell summaries, reservoir-linked analyses, proteomic overlays, evidence tables, and figure-source registers are supplied in the supporting information.

The revised manuscript now identifies the permanent Zenodo repository for author-generated analysis code and derived reproducibility materials: https://doi.org/10.5281/zenodo.22396354.

J03. Amended Funding Statement and Role of Funders

Response

I confirm that this study received no specific funding. My commercial affiliation with AfroBiomics Co. Ltd. is declared, but AfroBiomics Co. Ltd. provided no salary support for this study and no study-specific financial, material, computing, software, administrative, publication, or other support. AfroBiomics Co. Ltd. had no role in study design, data collection, analysis, interpretation, decision to publish, or manuscript preparation.

Funding Statement

The author received no specific funding for this work. Reuben S. Maghembe is affiliated with AfroBiomics Co. Ltd.; however, AfroBiomics Co. Ltd. provided no salary, financial, research-material, computing, software, administrative, publication, or other material support for this study and had no role in study design, data collection and analysis, interpretation, decision to publish, or preparation of the manuscript.

J04. Updated Competing Interests Statement

Response

I confirm my commercial affiliation as Chief Executive Officer of AfroBiomics Co. Ltd. AfroBiomics Co. Ltd. provided no financial or material support for this study and had no role in study design, data collection, analysis, interpretation, decision to publish, or manuscript preparation. I have no other competing interests to declare, and AfroBiomics Co. Ltd. imposes no restrictions on sharing the study's data, code, materials, or publication.

Competing Interests Statement

I am Chief Executive Officer of AfroBiomics Co. Ltd. I have no other competing interests to declare. This commercial affiliation provided no financial or material support for this study and had no role in study design, data collection, analysis, interpretation, decision to publish, or manuscript preparation. AfroBiomics Co. Ltd. imposes no restrictions on sharing the study's data, code, materials, or publication. This does not alter my adherence to PLOS ONE policies on sharing data and materials.

J05. Reviewer-recommended references

Response

I evaluated the four publications specifically recommended by Reviewer 2. They are relevant to the revised discussion of CH25H-mediated restriction, TRIM22-mediated restriction, dendritic-cell antiviral programs, and interferon-stimulated effector networks. They were incorporated through the protected Zotero-preserving reference-integration workflow at the relevant manuscript locations.

The requested publications are identified by PMIDs 23273844, 21345949, 31244850, and 31283436. The bibliographic record associated with PMID 21345949 also has a later correction record, PMID 39868830, which was handled during reference integration as bibliographic provenance without treating it as an additional reviewer-requested citation.

Reviewer 1

R1.01. Innate sensing and cell-death axis

Comment addressed

The reviewer noted that the original framework did not adequately expose the HIV-relevant sensing and cell-death axis, including IFI16, STING, TREX1, caspase-1/inflammasome biology, caspase-3, and the absence of cGAS from the Results.

Response

I agree that this axis required more explicit treatment. cGAS and STING were not actually absent from the prespecified framework: cGAS/MB21D1 and STING/TMEM173 were already represented in the interferon/innate-sensing module. I nevertheless added a focused analysis that makes these genes explicit and added IFI16, TREX1, CASP1, and CASP3 as clearly labeled reviewer-requested exploratory targets rather than retrospectively redefining the prespecified modules.

In GSE157198, TREX1 showed clear FDR-supported variation, whereas the bulk cGAS and STING contrasts were not FDR-supported. In GSE256089, IFI16, STING/TMEM173, and CASP3 were higher in NK cells, whereas TREX1 and CASP1 were higher in monocytes; cGAS/CGAS did not show a material NK-versus-monocyte difference.

I have explicitly bounded the interpretation. Transcript abundance does not demonstrate cGAS-STING pathway activation; CASP1 expression does not demonstrate inflammasome activation or pyroptosis; and CASP3 expression does not demonstrate executioner-caspase activation or apoptosis. The revised manuscript therefore presents these findings as expression-state observations and mechanistic hypotheses rather than functional cell-death measurements.

R1.02. Formal statistics for the coupling claims

Comment addressed

The reviewer requested quantitative statistical support for the core-restrictome coupling claims.

Response

I added formal sample-level coupling statistics in GSE157198. Core-restrictome and interferon/innate-sensing scores were strongly correlated (Spearman r=0.799718, p=7.46724×10^-11). Core-restrictome and lipid/cholesterol scores were also correlated in the unadjusted analysis (Spearman r=0.451163, p=0.00211408).

These values are now stated directly in the Results and Fig 2 legend rather than describing coupling qualitatively.

R1.03. Whether lipid coupling is independent of interferon

Comment addressed

The reviewer asked whether the core-restrictome/lipid association persists independently of the shared interferon axis.

Response

I performed two sensitivity analyses. First, removing CH25H, which overlaps the core and lipid/cholesterol modules, still gave an unadjusted association (Spearman r=0.424242, p=0.00410444). However, the association disappeared when interferon was controlled: partial Spearman r=0.059104, p=0.703132 after conditioning on IFN, and r=-0.017597, p=0.909734 after conditioning on both IFN and clinical group.

I therefore revised the interpretation throughout the manuscript. Lipid/cholesterol activity is no longer described as an independently coupled restrictome program. Instead, the data support a shared IFN-associated immunometabolic architecture in this cohort.

R1.04. NK-cell lineage baseline and specificity

Comment addressed

The reviewer noted that the original observation that NK cells had the highest score could reflect constitutive lineage-specific expression rather than specific restrictome enrichment.

Response

I agree that simple rank ordering was insufficient. I therefore moved the analysis to the participant level and compared each participant's NK core-restrictome score with that participant's mean score across B cells, monocytes, and T cells. Across 38 participants, the mean NK-minus-reference difference was 0.105124, with dz=3.8490 and paired p=5.551754×10^-24. NK cells had the highest score in 35 of 38 participants.

More importantly, I compared the observed effect with 1,000 random gene sets per prespecified matching scheme, controlling for gene-set size and expression/detection structure. The NK-versus-reference enrichment remained unusual under these matched nulls, with empirical upper-tail p values of 0.001998, 0.007992, and 0.000999 across the frozen matching schemes.

I have therefore changed the interpretation. The revised manuscript describes the NK pattern as constitutive/developmental antiviral readiness together with specific enrichment of the curated restrictome beyond matched background gene sets. It does not interpret the NK signal as uniform HIV-induced activation.

All inferential comparisons use participants, not cells, as independent units.

R1.05. Monocyte subset composition

Comment addressed

The reviewer requested evaluation of whether the bulk-monocyte clinical-state result could be driven by monocyte subset composition.

Response

I addressed this in two complementary ways.

First, in GSE256089, I compared participant-paired CD14 and FCGR3A monocyte subtypes. Across 38 participants, there were 37,081 CD14 cells and 7,581 FCGR3A cells. The core-restrictome score was higher in FCGR3A than CD14 monocytes by 0.157650 on average (dz=2.95165; paired p=4.773127×10^-20), with FCGR3A exceeding CD14 in 37 of 38 participants. Monocyte composition was therefore biologically associated with restrictome state.

Second, I tested three transcriptional proxies of CD14-FCGR3A balance in the independent GSE157198 bulk-monocyte cohort. Adjustment for these proxies left the ART-naive clinical-state effect essentially unchanged at approximately 1.14–1.17, with p values approximately 0.0006–0.0011.

The revised interpretation is:

Monocyte subtype composition is biologically associated with restrictome activity, but transcriptional proxies of CD14-FCGR3A balance did not account for the clinical-state-associated restrictome elevation observed in the independent GSE157198 bulk-monocyte cohort.

R1.06. Mechanistic and translational interpretation of Fig 6

Comment addressed

The reviewer considered the architecture useful but requested an explicit mechanistic hypothesis and translational interpretation.

Response

I revised Fig 6 around three deliberately separated levels:

1. observed evidence;

2. noncausal mechanistic hypotheses; and

3. perturbational tests.

The figure no longer presents arrows as established causal pathways. Instead, the observed transcriptomic, cell-localization, and proteomic findings motivate testable hypotheses concerning IFN state, immune-cell lineage, lipid/cholesterol state, nucleotide availability, proteostasis/autophagy, and redox context.

The revised Discussion specifies perturbational tests such as manipulating cholesterol synthesis, nucleotide availability, redox state, or autophagy/proteostasis while measuring both restriction-factor abundance and antiviral function in defined immune-cell compartments. The translational implication is correspondingly bounded: host-directed interventions should be evaluated in the cell type and molecular layer in which the relevant restriction program operates rather than assumed to act uniformly across immune compartments.

R1.07. Title and prose complexity

Comment addressed

The reviewer recommended a simpler title and clearer wording.

Response

I agree and have substantially simplified the title and manuscript-wide prose.

The revised title is:

Cross-omics mapping of the human HIV restrictome across clinical states, immune-cell compartments, and protein responses

I also replaced several abstract and Introduction formulations that could imply a stronger integrated or causal model than the data support. The revised framing states that the data suggest context-specific combinations of antiviral defenses across clinical states and immune-cell compartments rather than a uniform intrinsic antiviral program.

Reviewer 2

R2.01. Random-gene-set / permutation robustness

Comment addressed

The reviewer requested null testing to establish that both the monocyte disease-state separation and NK-cell enrichment were not generic transcriptomic background effects.

Response

I addressed the two parts separately because they involve different experimental structures.

Bulk monocyte disease-state signal

The GSE157198 robustness analysis used three prespecified null schemes, with 1,000 random 37-gene sets per scheme (3,000 total). The observed ART-naive-minus-healthy core-restrictome difference was 1.153725 (Welch p=0.000745751) and was more extreme than the null distribution in all three schemes (empirical p=0.000999001 in each; BH-adjusted empirical p=0.002997 in each).

The observed core-IFN correlation (Spearman r=0.799718) was also highly unusual under the null schemes, with empirical p values of 0.000999001, 0.001998002, and 0.000999001 and corresponding BH-adjusted values of 0.002997, 0.004995, and 0.002997. By contrast, the unadjusted core-lipid relationship was less stable under expression-matched nulls (empirical p=0.0239760, 0.0989011, and 0.0929071), and the IFN-adjusted core-lipid association remained null under all schemes (empirical p=0.709291, 0.700300, and 0.715285). These results support the specificity and robustness of the disease-state and core-IFN signals while reinforcing the revised conclusion that the lipid association is not independent of interferon.

NK-cell enrichment

For the NK analysis, I generated 1,000 size- and expression/detection-matched random gene sets per prespecified null scheme. The observed participant-level NK enrichment exceeded the matched background distributions, with frozen empirical upper-tail p values of 0.001998, 0.007992, and 0.000999 across the relevant matching schemes.

Thus, the revised manuscript no longer relies on the observation that NK cells simply rank highest.

R2.02. Leave-one-gene-out analysis of the 37-gene core restrictome

Comment addressed

The reviewer requested a jackknife analysis to determine whether one dominant gene drives the GSE157198 clinical-state effect.

Response

I performed 37 leave-one-core-gene-out analyses. The ART-naive-versus-healthy disease effect retained the same direction in 37/37 jackknife analyses. This supports a distributed module-level signal rather than an effect dependent on one dominant member of the core restrictome.

The result is now stated in the Results and Fig 2 legend.

R2.03. Higher-resolution NK and monocyte subtypes

Comment addressed

The reviewer suggested CD56dim versus CD56bright NK cells and CD14-positive versus CD16-positive monocytes where the data support such analysis.

Response

I performed the higher-resolution analysis only where valid labels existed.

For monocytes, the historical single-cell annotations supported CD14 and FCGR3A subtypes, enabling participant-paired analysis. FCGR3A core-restrictome scores exceeded CD14 scores in 37 of 38 participants, with a mean difference of 0.157650 and paired p=4.773127×10^-20.

For NK cells, the single-cell dataset did not contain validated participant-linked CD56bright and CD56dim single-cell labels. Although separate flow-cytometry metadata contained CD56dim information, it did not establish matched single-cell CD56bright/CD56dim assignments suitable for the requested transcriptomic comparison. I therefore did not manufacture an NK-subtype classification from marker assumptions that were not validated in the source dataset.

The limitation is now stated explicitly.

R2.04. Missing-value imputation in PXD012263

Comment addressed

The reviewer requested clarification of missing-value handling.

Response

I traced the complete time-course overlay and polarity workflow to the source tables and adjudicated every imputation-related operation. No quantitative protein-abundance imputation was identified in this reanalysis. Missing source-table abundance values were retained as missing.

Replicate summaries used available nonmissing source values, and a pseudocount of 1 was included in derived log2-ratio calculations. The pseudocount did not replace missing protein-abundance measurements and is therefore distinct from missing-value imputation. The identified fill operations were likewise non-abundance operations: fillna(0) was used for a derived protein-priority score, and fillna(99) was used for categorical sorting order.

I have revised the Methods to state this explicitly.

R2.05. FDR-threshold sensitivity

Comment addressed

The reviewer requested evaluation beyond q<0.05.

Response

I evaluated the existing published q values at q<0.01, q<0.05, and q<0.10. Among 137 q-evaluable curated genes, the thresholds selected 10, 19, and 25 genes, respectively. The q<0.05 analysis reproduced the historical 19-gene membership exactly.

Mixed protein-response polarity persisted across the thresholds, although the relative balance of increased, depleted, and stable/subtle proteins varied with threshold. I therefore retain historical q<0.05 as the primary proteomic analysis and present q<0.01 and q<0.10 strictly as sensitivity analyses.

The time-course abundance panels remain descriptive and are not presented as independent differential-significance tests.

R2.06. Terminology and study type

Comment addressed

The reviewer recommended distinguishing the study from a clinical PRISMA meta-analysis.

Response

I agree. The manuscript now consistently uses the term:

“evidence-structured computational reanalysis of public datasets”

The three datasets are described as complementary evidence layers. They are not described as a subject-matched multi-omics cohort, and the manuscript does not imply within-subject cross-platform trajectories.

R2.07. Constitutive antiviral priming of NK cells

Comment addressed

The reviewer asked that the Discussion acknowledge the constitutive antiviral readiness of NK cells.

Response

I added this distinction to the revised Discussion. The high NK core-restrictome score is interpreted as a combination of lineage/developmental antiviral readiness and specific curated-restrictome enrichment beyond size- and expression/detection-matched null gene sets.

I explicitly avoid interpreting the broad NK signal as evidence that HIV uniformly induced the program in NK cells.

R2.08. Recommended references

Comment addressed

The reviewer recommended four publications concerning CH25H, TRIM22, dendritic-cell programs, and ISG effector networks.

Response

I evaluated all four and consider them relevant to the revised biological interpretation. They were incorporated through the protected Zotero-preserving reference-integration workflow at the relevant manuscript locations:

- PMID 23273844 — CH25H and viral-entry restriction;

- PMID 21345949 — TRIM22-mediated HIV transcriptional restriction;

- PMID 31244850 — dendritic-cell antiviral programs;

- PMID 31283436 — interferon-stimulated effector networks.

The later correction record PMID 39868830 was handled as bibliographic provenance for PMID 21345949 rather than as a fifth reviewer-requested reference.

Final reference numbering was generated through Zotero during the protected reference-integration stage.

R2.09. Textual and editorial revisions

Response

I simplified the title, Abstract, Introduction conclusion, Results interpretation, Discussion, and figure legends. I also standardized the study description as an evidence-structured computational reanalysis and maintained single-author wording throughout.

The revised text avoids claims of subject-matched multi-omics, avoids causal language unsupported by the datasets, and distinguishes observed expression from pathway activation and cell-death mechanisms.

R2.11. Figure cleanup and accessibility

Comment addressed

The reviewer requested removal of working notes, correction of text collisions, improved box centering, consistent typography, and consideration of color accessibility.

Response

I addressed these presentation issues in the final frozen figure set. The revised versions remove the working annotations, resolve the identified collisions, improve text placement and typography, and retain the scientifically approved panel architecture.

No scientific model was refitted and no figure-level statistical analysis was rerun merely for presentation cleanup.

Closing response

I appreciate the reviewers' suggestions. The additional analyses materially strengthened the manuscript by distinguishing robust clinical-state and NK signals from background structure, separating lipid/cholesterol association from the shared interferon axis, identifying a monocyte-subtype contribution without attributing the independent bulk disease effect to composition, and more clearly bounding the mechanistic interpretation of the sensing/death and proteomic results.

The revised manuscript now presents the human HIV restrictome as a context-dependent pattern observed across independent public evidence layers, with explicit separation between observed associations, mechanistic hypotheses, and future perturbational tests.

Sincerely,

Reuben S. Maghembe

Attachment Submitted filename: Response_to_Reviewers_PONE-D-26-39263_cross_omics.docx

# Peer review: Decision Letter 1

**DOI:** 10.1371/journal.pone.0356591.r004

**Authors:** Carlos de Noronha

22 Sep 2026

Cross-omics mapping of the human HIV restrictome across clinical states, immune-cell compartments, and protein responses

PONE-D-26-39263R1

Dear Dr. Maghembe,

We’re pleased to inform you that your manuscript has been judged scientifically suitable for publication and will be formally accepted for publication once it meets all outstanding technical requirements.

Within one week, you’ll receive an e-mail detailing the required amendments. When these have been addressed, you’ll receive a formal acceptance letter and your manuscript will be scheduled for publication.

An invoice will be generated when your article is formally accepted. Please note, if your institution has a publishing partnership with PLOS and your article meets the relevant criteria, all or part of your publication costs will be covered. Please make sure your user information is up-to-date by logging into Editorial Manager at Editorial Manager® and clicking the ‘Update My Information' link at the top of the page. For questions related to billing, please contact billing support.

If your institution or institutions have a press office, please notify them about your upcoming paper to help maximize its impact. If they’ll be preparing press materials, please inform our press team as soon as possible -- no later than 48 hours after receiving the formal acceptance. Your manuscript will remain under strict press embargo until 2 pm Eastern Time on the date of publication. For more information, please contact onepress@plos.org.

Kind regards,

Carlos Manuel de Noronha, Sc.D.

Academic Editor

PLOS One

Additional Editor Comments (optional):

Reviewers' comments:

Reviewer's Responses to Questions

**Comments to the Author**

1. If the authors have adequately addressed your comments raised in a previous round of review and you feel that this manuscript is now acceptable for publication, you may indicate that here to bypass the “Comments to the Author” section, enter your conflict of interest statement in the “Confidential to Editor” section, and submit your "Accept" recommendation.

Reviewer #2: (No Response)

**********

2. Is the manuscript technically sound, and do the data support the conclusions?

The manuscript must describe a technically sound piece of scientific research with data that supports the conclusions. Experiments must have been conducted rigorously, with appropriate controls, replication, and sample sizes. The conclusions must be drawn appropriately based on the data presented.

Reviewer #2: (No Response)

**********

3. Has the statistical analysis been performed appropriately and rigorously?

Reviewer #2: (No Response)

**********

4. Have the authors made all data underlying the findings in their manuscript fully available?

The PLOS Data policy requires authors to make all data underlying the findings described in their manuscript fully available without restriction, with rare exception (please refer to the Data Availability Statement in the manuscript PDF file). The data should be provided as part of the manuscript or its supporting information, or deposited to a public repository. For example, in addition to summary statistics, the data points behind means, medians and variance measures should be available. If there are restrictions on publicly sharing data—e.g. participant privacy or use of data from a third party—those must be specified.

Reviewer #2: (No Response)

**********

5. Is the manuscript presented in an intelligible fashion and written in standard English?

PLOS ONE does not copyedit accepted manuscripts, so the language in submitted articles must be clear, correct, and unambiguous. Any typographical or grammatical errors should be corrected at revision, so please note any specific errors here.

Reviewer #2: (No Response)

**********

6. Review Comments to the Author

Please use the space provided to explain your answers to the questions above. You may also include additional comments for the author, including concerns about dual publication, research ethics, or publication ethics. (Please upload your review as an attachment if it exceeds 20,000 characters)

Reviewer #2: (No Response)

**********

7. PLOS authors have the option to publish the peer review history of their article (what does this mean?). If published, this will include your full peer review and any attached files.

If you choose “no”, your identity will remain anonymous but your review may still be made public.

**Do you want your identity to be public for this peer review?**  For information about this choice, including consent withdrawal, please see our Privacy Policy.

Reviewer #2: No

**********

# Editor report: Acceptance letter

**DOI:** 10.1371/journal.pone.0356591.r005

**Authors:** Carlos de Noronha

PONE-D-26-39263R1

PLOS One

Dear Dr. Maghembe,

I'm pleased to inform you that your manuscript has been deemed suitable for publication in PLOS One. Congratulations! Your manuscript is now being handed over to our production team.

At this stage, our production department will prepare your paper for publication. This includes ensuring the following:

* All references, tables, and figures are properly cited

* All relevant supporting information is included in the manuscript submission,

* There are no issues that prevent the paper from being properly typeset

You will receive further instructions from the production team, including instructions on how to review your proof when it is ready. Please keep in mind that we are working through a large volume of accepted articles, so please give us a few days to review your paper and let you know the next and final steps.

Lastly, if your institution or institutions have a press office, please let them know about your upcoming paper now to help maximize its impact. If they'll be preparing press materials, please inform our press team within the next 48 hours. Your manuscript will remain under strict press embargo until 2 pm Eastern Time on the date of publication. For more information, please contact onepress@plos.org.

You will receive an invoice from PLOS for your publication fee after your manuscript has reached the completed accept phase. If you receive an email requesting payment before acceptance or for any other service, this may be a phishing scheme. Learn how to identify phishing emails and protect your accounts at https://explore.plos.org/phishing.

If we can help with anything else, please email us at customercare@plos.org.

Thank you for submitting your work to PLOS One and supporting open access.

Kind regards,

PLOS One Editorial Office Staff

on behalf of

Dr. Carlos Manuel de Noronha

Academic Editor

PLOS One
